# ﻿An overview of Melanommataceae (Pleosporales, Dothideomycetes): Current insight into the host associations and geographical distribution with some interesting novel additions from plant litter

**DOI:** 10.3897/mycokeys.106.125044

**Published:** 2024-06-17

**Authors:** Danushka S. Tennakoon, Kasun M. Thambugala, Nimali I. de Silva, Hai-Yan Song, Nakarin Suwannarach, Fu-Sheng Chen, Dian-Ming Hu

**Affiliations:** 1 Bioengineering and Technological Research Centre for Edible and Medicinal Fungi, Jiangxi Agricultural University, Nanchang 330045, China; 2 Nanchang Key Laboratory of Edible and Medicinal Fungi, Jiangxi Agricultural University, Nanchang 330045, China; 3 Jiangxi Provincial Key Laboratory of Subtropical Forest Resource Cultivation, Jiangxi Agricultural University, Nanchang 330045, China; 4 Genetics and Molecular Biology Unit, Faculty of Applied Sciences, University of Sri Jayewardenepura, Gangodawila, Nugegoda 10250, Sri Lanka; 5 Center for Biotechnology, Department of Zoology, University of Sri Jayewardenepura, Nugegoda 10250, Sri Lanka; 6 Center for Plant Materials and Herbal Product Research, Department of Botany, Faculty of Applied Sciences, University of Sri Jayewardenepura, Nugegoda 10250, Sri Lanka; 7 Center of Excellence in Microbial Diversity and Sustainable Utilization, Chiang Mai University, Chiang Mai 50200, Thailand; 8 Department of Biology, Faculty of Science, Chiang Mai University, Chiang Mai, 50200, Thailand

**Keywords:** Biodiversity, multi-gene phylogeny, new host records, Pleosporales, saprobes, systematics

## Abstract

Melanommataceous species exhibit high diversity with a cosmopolitan distribution worldwide and show a prominent saprobic lifestyle. In this study, we explored five saprobic species collected from plant litter substrates from terrestrial habitats in China and Thailand. A combination of morphological characteristics and multi-locus phylogenetic analyses was used to determine their taxonomic classifications. Maximum Likelihood and Bayesian Inference analyses of combined LSU, SSU, ITS and *tef1-α* sequence data were used to clarify the phylogenetic affinities of the species. *Byssosphaeriapoaceicola* and *Herpotrichiazingiberacearum* are introduced as new species, while three new host records, *Bertiellafici*, *By.siamensis* and *Melanommapopulicola* are also reported from litter of *Cinnamomumverum*, *Citrustrifoliata* and *Fagussylvatica*, respectively. Yet, despite the rising interest in the melanommataceous species, there is a considerable gap in knowledge on their host associations and geographical distributions. Consequently, we compiled the host-species associations and geographical distributions of all the so far known melanommataceous species.

## ﻿Introduction

Fungi occur in a wide range of ecosystems (Hawksworth 2001; De Silva et al. 2016; Hernández-Restrepo et al. 2017). With over 98,334 extant species, Ascomycota is the largest phylum of fungi and is widely distributed in terrestrial, freshwater and marine environments (Hill et al. 2021; Senanayake et al. 2022; Bánki et al. 2023). The class Dothideomycetes is estimated to have 32,365 species and is one of the most ecologically diverse group of ascomycetes (Hongsanan et al. 2020; Bánki et al. 2023). Pleosporales is considered the largest and most diverse order in the class Dothideomycetes (Zhang et al. 2012; Hongsanan et al. 2020). They exhibit a wide range of lifestyles (e.g. biotrophs, endophytes, epiphytes, hemibiotrophs, pathogens, saprobes) and can be found worldwide, including terrestrial, marine and freshwater environments (Jones et al. 2019; Hongsanan et al. 2020; Calabon et al. 2022; Gao et al. 2023). In addition, they are extremely adaptable to various ecological niches and can exist in anaerobic, aquatic, mutualistic, terrestrial and even in severe habitats, such as deserts (Zhang et al. 2012; Hyde et al. 2013; Hongsanan et al. 2020). Pleosporales species play significant functional roles from an agricultural, ecological and economic perspectives (Zhang et al. 2012; Raja et al. 2017; Wen et al. 2020; Pimenta et al. 2021). According to the recent outline of Wijayawardene et al. (2022), Pleosporales consists of 91 families.

Melanommataceae is one of the species-rich families in Pleosporales, Dothideomycetes. It was introduced by Winter (1885) to include *Melanomma* as the type genus and species have globose or depressed ascomata, fissitunicate asci, pigmented and phragmosporous ascospores (Tian et al. 2015; Hongsanan et al. 2020). The ordinal level placement of Melanommataceae was controversial for a long time and Barr (1983) introduced Melanommatales to accommodate all taxa which have trabeculate pseudoparaphyses. The nature of trabeculate pseudoparaphyses was broadly discussed by Liew et al. (2000) and illustrated that they are generally 1 μm or thinner, clearly anastomose between the asci and are embedded in a gelatinous matrix. Subsequently, Barr (1990) introduced five genera in Melanommataceae (*Byssosphaeria*, *Keissleriella*, *Melanomma*, *Ostropella* and *Strickeria*) based on erumpent to superficial ascomata and thick-walled peridium. Over time, Melanommataceae has been transferred from Melanommatales to Pleosporales with the revolution of DNA-based molecular phylogenetic studies (Eriksson 2006; Wang et al. 2007; Zhang et al. 2012; Hyde et al. 2013; Tian et al. 2015; Hongsanan et al. 2020).

The species of Melanommataceae have cosmopolitan distribution worldwide in temperate, subtropical and tropical regions (Hyde et al. 2013; Tian et al. 2015; Jaklitsch and Voglmayr 2017; Kularathnage et al. 2022). They can play a vital role as saprobes, endophytes or hyperparasites and occur on twigs or bark of various woody plants in terrestrial, marine or freshwater habitats (Tian et al. 2015; Hashimoto et al. 2017; Tennakoon et al. 2018; Hongsanan et al. 2020; Gao et al. 2023). In addition, some species (e.g. *Seifertiaalpina*) have been recorded as plant pathogens and cause bud blight or bud blast disease of *Rhododendron* species (Glawe and Hummel 2006; Li et al. 2016a). Interestingly, several species have also been reported from soil (e.g. *Herpotrichiagelasinosporoides*, *H.striatispora* and *Pleotrichocladiumopacum*), on lichen species (e.g. *Aposphaeriaramalinae*) and on mushroom species (e.g. *Exosporiellafungorum* on *Thelephora* sp.) (Karsten 1892; Pitard and Harmand 1911).

**Figure 1.**
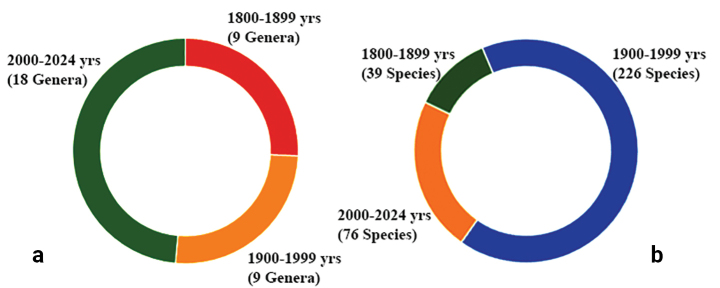
**a** The number of melanommataceous genera introduced in different time periods **b** the number of melanommataceous species introduced in different time periods (Source – MycoBank Database).

**Figure 2.**
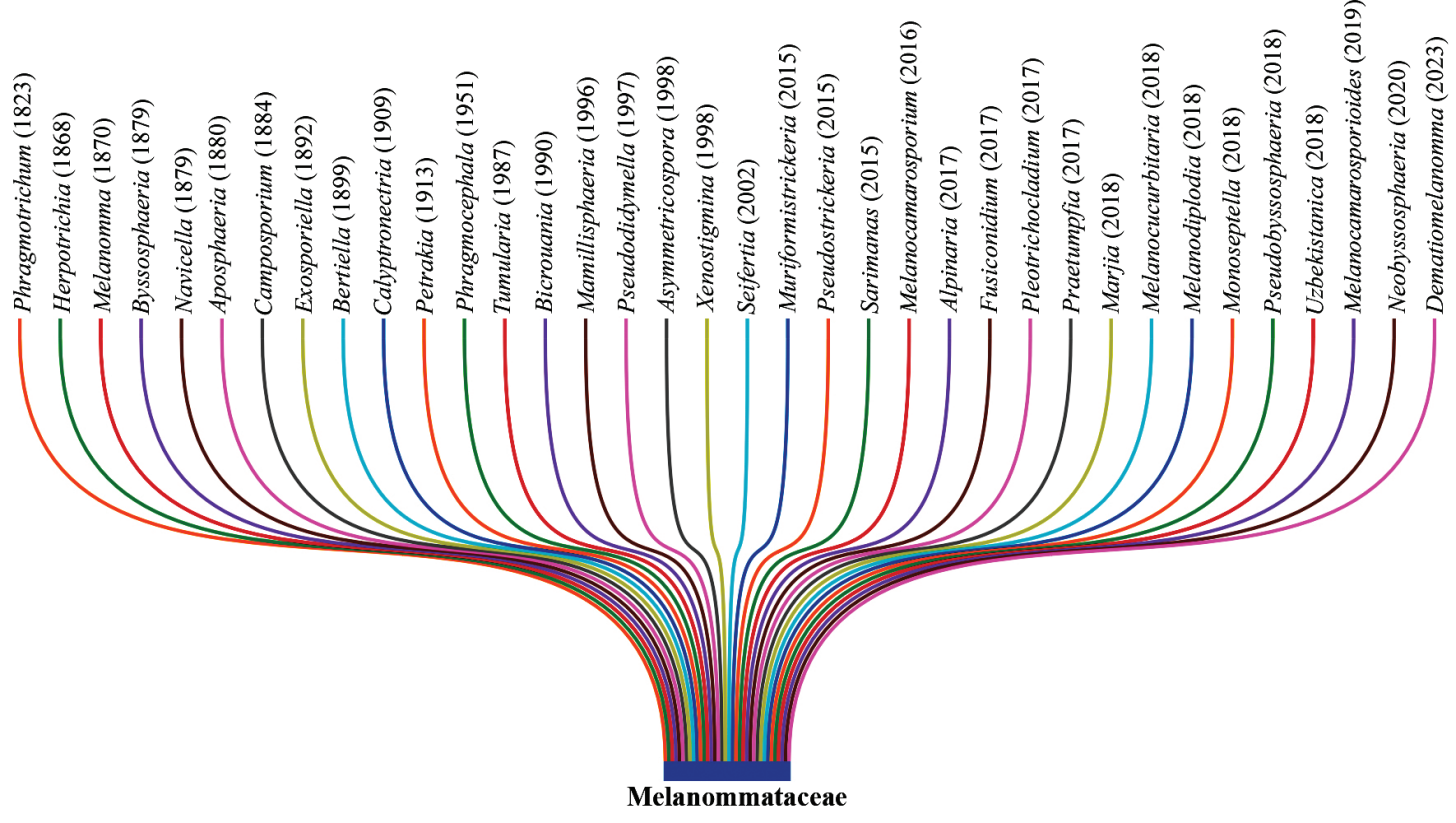
The melanommataceous genera introduced in different time periods (Source – MycoBank Database).

The cosmopolitan nature of Melanommataceae is further supported by the numerous novel genera and species that have been introduced in the past years. Based on the year of introduction, we compiled the data and revealed that nine genera were introduced between 1800 and 1899 and nine more genera between 1900 and 1999. In addition, 18 genera were introduced between 2000 and 2024 (Fig. 1). This rapid increase may be primarily due to the revolution in fungal taxonomical studies with DNA sequence-based molecular phylogenies in the last two decades. Interestingly, six genera were introduced in the year 2018, such as *Marjia*, *Melanocucurbitaria*, *Melanodiplodia*, *Monoseptella*, *Pseudobyssosphaeria* and *Uzbekistanica* (Fig. 2). Consequently, 36 genera are currently accepted in Melanommataceae, viz. *Alpinaria*, *Aposphaeria*, *Asymmetricospora*, *Bertiella*, *Bicrouania*, *Byssosphaeria*, *Calyptronectria*, *Camposporium*, *Dematiomelanomma*, *Exosporiella*, *Fusiconidium*, *Herpotrichia*, *Mamillisphaeria*, *Marjia*, *Melanocamarosporioides*, *Melanocamarosporium*, *Melanocucurbitaria*, *Melanodiplodia*, *Melanomma*, *Monoseptella*, *Muriformistrickeria*, *Navicella*, *Neobyssosphaeria*, *Petrakia*, *Phragmocephala*, *Phragmotrichum*, *Pleotrichocladium*, *Praetumpfia*, *Pseudobyssosphaeria*, *Pseudodidymella*, *Pseudostrickeria*, *Sarimanas*, *Seifertia*, *Tumularia*, *Uzbekistanica* and *Xenostigmina* (Hongsanan et al. 2020; Gao et al. 2023). Conversely, the species discoveries are much higher during the 1900 and 1999 period (226 species) and thirty-nine species have been introduced between 1800 and 1899. However, despite having introduced 18 genera during the 2000–2024 period, only 76 species have been introduced (Fig. 1). Nevertheless, it could be much more in future with the extensive taxon samplings, particularly in poorly studied countries/regions, substrates and hosts.

We are exploring the fungal diversity of plant litter substrates with the aim of clarifying their taxonomy, based on morphology coupled with multi-gene phylogeny (Thambugala et al. 2017; Tennakoon et al. 2018, 2021, 2023; Wanasinghe et al. 2018). Thus, we have collected five taxa from China and Thailand which belong to the family Melanommataceae. The objectives of this study are to identify the melanommataceous taxa associated with plant litter using both morphological and phylogenetic approaches and to provide an updated checklist of species in Melanommataceae. This study provides a database on melanommataceous species for future studies, increases knowledge of fungal diversity and helps to understand their global distribution and host associations.

## ﻿Materials and methods

### ﻿Sample collection and examination

Fresh fungal specimens were collected from plant litter (dead wood and leaves) from Chiang Mai, Thailand and Kunming, China. The collected specimens were taken back to the laboratory in zip lock bags and paper envelopes. All the samples were subjected for an incubation period (one day) in plastic boxes lined with wet tissue paper. The micro- and macro-morphological characteristics were observed as described by Tennakoon et al. (2023). Sections of ascomata were taken manually and were mounted in distilled water. A stereomicroscope (AXIOSKOP 2 PLUS Series, Göttingen, Germany) was used to examine the surface morphological characteristics of fungal fruiting bodies. Micro-morphological characteristics, such as asci, ascospores and pseudoparaphyses were examined using Axioskop 2 Plus (Göttingen, Germany) compound microscope. Images were taken using Canon Axiocam 506 color digital camera (Hanover, Germany) fitted to a Axioskop 2 Plus (Göttingen, Germany) compound microscope. The micro morphological characteristics, such as colour, shape, height and diameter of ascomata, asci, ascospores, peridium and pseudoparaphyses were recorded. Indian ink was used to inspect the existence of the mucilaginous sheath in ascospores. The prepared slides were permanently preserved using lactoglycerol and sealed by applying nail-polish around the margins of cover slips. All measurements were obtained using Tarosoft (R) Image Framework application. Adobe Photoshop CS3 Extended version 10.0 software (Adobe Systems, USA) was used to construct the photo plates. Specimens were deposited in the Herbarium of Department of Biology (CMUB) and Sustainable Development of Biological Resources Laboratory (SDBR), Faculty of Science, Chiang Mai University and Herbarium of Fungi, Jiangxi Agricultural University (HFJAU), Nanchang, China. The Faces of Fungi (FOF) and Index Fungorum (IF) numbers were obtained for new species (*Byssosphaeriapoaceicola* and *Herpotrichiazingiberacearum*) as mentioned in Jayasiri et al. (2015) and Index Fungorum (2024).

### ﻿DNA extraction, PCR amplification and sequencing

Genomic DNA was extracted from fungal fruiting bodies on the natural substrate by using a DNA extraction kit (E.Z.N.A. ® Forensic DNA Kit, D3591-01, Omega BIO-TEK) following the manufacturer’s protocol. DNA products were intended for use as a template for PCR and stored at 4 °C and the duplicates were kept at -20 °C for long-term storage. Four genomic regions were amplified, the internal transcribed spacer (ITS) region (ITS1-5.8S-ITS2), the 28S large subunit rDNA (LSU), 18S small subunit rDNA (SSU) and the translation elongation factor 1-alpha gene (tef1-α). The primers ITS4 and ITS5 were used to amplify the ITS (White et al. 1990), LR0R and LR5 primers for LSU (Vilgalys and Hester 1990), NS1 and NS4 for SSU (White et al. 1990) and EF1-983F and EF1-2218R primers for *tef1-α* (Rehner 2001). The amplification reactions were performed in a total reaction volume of 25 µl, which contained 9.5 µl of sterilised distilled water, 12.5 µl of 2 × Power Taq PCR MasterMix (a premix and ready to use solution, including 0.1 Units/μl Taq DNA Polymerase, 500 μM dNTP Mixture each (dATP, dCTP, dGTP, dTTP) (Bioteke Co., China), 1 μl of each forward and reverse primers (stock concentration 10 pM) and 1 μl of DNA template. The polymerase chain reaction (PCR) thermal cycle programmes for ITS, LSU, SSU and *tef1-α* genes amplification were adjusted as described in Tennakoon et al. (2022). To check the quality of PCR products, agarose gel electrophoresis (1%) was conducted. The purified PCR products were subjected for sequencing at Sangon Biotech (Shanghai) Co., Ltd, China. Generated sequences were deposited in GenBank and accession numbers were listed (Table 1).

**Table 1.** GenBank and culture collection accession numbers of species included in the phylogenetic study. The newly-generated sequences are shown in bold face.

Fungal Species	Strain/Voucher No.	GenBank Accession Number			
		ITS	LSU	SSU	*tef*–1α
*Alpinariarhododendri*	KT 2520	LC203335	LC203360	LC203314	LC203388
*Aposphaeriacorallinolutea*	MFLU 15-2752	KY554202	KY554197	KY554200	KY554205
*A.corallinolutea*	GLMC 1355	MT153708	MT156159	–	–
*Bertiellaellipsoidea*	MFLU 16-0583	KX765261	KX765262	–	–
*B.ellipsoidea*	MFLUCC 17-2015	MG543922	MG543913	–	–
*B.fici*	MFLUCC 20-0229	–	MW063223	MW079351	MW183786
*B.fici*	NCYUCC 19-0260	–	MW063224	MW079352	MW183787
*B.fici*	NCYUCC 19-0290	–	MW063225	MW079353	MW183788
***B.fici***	**CMUB 40045**	–	**PP460772**	**PP460764**	**PP475453**
*B.macrospora*	IL 5005	–	GU385150	–	–
*B.macrospora*	SMH 3953	–	–	–	GU327744
*Beverwykellapulmonaria*	CBS 283.53	KY189974	GU301804	–	–
*Byssosphaeriadiffusa*	AFTOL ID 1588	–	DQ678071	DQ678019	DQ677915
*By.diffusa*	CBS 250.62	–	–	GU205239	–
*By.jamaicana*	SMH 1403	–	GU385152	–	GU327746
*By.jamaicana*	SMH 3085	–	GU385154	–	–
*By.jamaicana*	SMH 3464	–	GU385153	–	–
*By.macarangae*	MFLUCC 17-2655	MH389782	MH389778	MH389780	MH389784
*By.musae*	MFLUCC 11-0146	KP744435	KP744477	KP753947	MH581149
*By.phoenicis*	ZHKUCC 21-0122	ON180685	ON180683	ON180691	ON243583
*By.phoenicis*	ZHKUCC 21-0123	ON180686	ON180684	ON180692	ON243584
*By.rhodomphala*	SMH3086	–	GU385155	–	–
*By.rhodomphala*	ANM 942	–	GU385160	–	–
*By.rhodomphala*	SMH 3402	–	GU385170	–	–
*By.rhodomphala*	GKM L153N	–	GU385157	–	GU327747
*By.salebrosa*	SMH 2387	–	GU385162	–	GU327748
*By.schiedermayeriana*	SMH 1816	–	GU385159	–	–
*By.schiedermayeriana*	SMH 1269	–	GU385158	–	–
*By.schiedermayeriana*	SMH 3157	–	GU385163	–	GU327745
*By.siamensis*	MFLUCC 10-0099	–	KT289895	KT289897	–
*By.siamensis*	MFLUCC 17-1800	MG543923	MG543914	MG543917	–
***By.siamensis***	**HFJAU10336**	**PP460780**	**PP460773**	**PP460765**	**PP475454**
*By.taiwanense*	MFLUCC 17-2643	MH389783	MH389779	MH389781	MH389785
*By.villosa*	GKM 204 N	–	GU385151	–	GU327751
***By.poaceicola***	**HFJAU10337**	**PP460781**	**PP460774**	**PP460766**	**PP475455**
***By.poaceicola***	**HFJAU10338**	**PP460782**	**PP460775**	**PP460767**	**PP475456**
*Fusiconidiumaquaticum*	KUMCC 15-0300	–	KX641894	KX641895	KX641896
*F.mackenziei*	MFLUCC 14-0434	–	KX611112	KX611114	KX611118
*Herpotrichiaherpotrichoides*	GKM 212N	–	GU385169	–	–
*H.herpotrichoides*	SMH 5167	–	GU385175	–	–
*H.macrotricha*	GKM 196N	–	GU385176	–	GU327755
*H.macrotricha*	SMH 269	–	GU385177	–	–
*H.macrotricha*	SMH 269	–	GU385177	–	GU327756
*H.vaginatispora*	MFLUCC 13-0865		KT934252	KT934256	KT934260
*H.xiaokongense*	KUMCC 21-0004	–	MZ408889	MZ408891	MZ394066
***H.zingiberacearum***	**HFJAU10332**	**PP460783**	**PP460776**	**PP460768**	**PP475457**
***H.zingiberacearum***	**HFJAU10333**	**PP460784**	**PP460777**	**PP460769**	**PP475458**
***H.zingiberacearum***	**HFJAU10334**	**PP460785**	**PP460778**	**PP460770**	**PP475459**
*Hysteriumangustatum*	MFLU 16-1179	KX611363	KX611364	KX611365	–
*Marjiatianschanica*	TASM 6120	MG828909	MG829019	MG829126	MG829206
*M.tianschanica*	TASM 6121	MG828910	MG829020	MG829127	MG829206
*Marjiauzbekistanica*	TASM 6122	MG828911	MG829021	MG829128	MG829208
*Melanocucurbitariauzbekistanica*	MFLUCC 17-0829	MG828912	MG829022	MG829129	MG829209
*Melanodiplodiatianschanica*	TASM 6111	MG828914	MG829023	MG829130	MG829210
*Me.tianschanica*	TASM 6112	MG828915	MG829024	MG829131	MG829211
*Me.tianschanica*	MFLUCC 17-0805	MG828913	MG829025	MG829132	MG829212
*Melanommajaponicum*	KT 3425	LC203320	LC203338	LC203292	LC203367
*Mel.populicola*	CBS 543.70	NR_170056	NG_075164	NG_070237	–
*Mel.populicola*	CPC 27203	MT223817	MT223910	–	–
*Mel.populicola*	CBS 350 82	MT223815	JF740265	–	–
*Mel.populicola*	CBS 130330	–	JF740328	–	–
***Mel.populicola***	**HFJAU10335**	**PP460786**	**PP460779**	**PP460771**	**PP475460**
*Mel.pulvis-pyrius*	KT 2110	LC203322	LC203340	LC203294	LC203368
*Mel.pulvis-pyrius*	KT 2113	LC203323	LC203341	LC203295	LC203369
*Mel.pulvis-pyrius*	AH 375	LC203324	LC203342	LC203296	LC203370
*Mel.pulvis-pyrius*	KH 27	LC203325	LC203343	LC203297	LC203371
*Mel.pulvis-pyrius*	CBS 124080	MH863349	GU456323	GU456302	GU456265
*Monoseptellarosae*	MFLUCC 17-0815	MG828916	MG829026	MG829133	MG829213
*Mo.rosae*	TASM 6114	MG828917	MG829027	MG829134	MG829214
*Mo.tuberculata*	CBS 256.84	–	GU301851	–	GU349006
*Muriformistrickeriarubi*	MFLUCC 15-0681	–	KT934253	KT934257	KT934261
*Petrakiairregularis*	CBS 306.67	NR_164281	MH870670	–	–
*Phragmocephalaatra*	MFLUCC 15-0021	KP698721	KP698725	KP698729	–
*P.garethjonesii*	MFLUCC 15-0018	KP698722	KP698726	KP698730	–
*Pleotrichocladiumopacum*	CBS 450.70	MH859791	KY853524	–	–
*Pl.opacum*	CBS 709.92	KY853464	KY853526	–	–
*Pseudostrickeriarosae*	MFLUCC 17-0643	MG828954	MG829065	MG829169	MG829234
*Ps.mutabilis*	SMH 1541	–	GU385209	–	–
*Sarimanaspseudofluviatile*	KT 760	LC001717	LC001714	LC001711	–
*S.shirakamiense*	KT 3000	–	LC001715	LC001712	–
*Seifertiaazaleae*	DAOM 239136	–	EU030276	–	–
*Se.shangrilaensis*	MFLUCC 16-0238	–	KU954100	KU954101	KU954102
*Uzbekistanicarosae-hissaricae*	MFLUCC 17-0819	MG828976	MG829087	MG829187	MG829242
*U.rosae-hissaricae*	MFLUCC 17-0820	GU269840	MG829088	MG829188	MG829243
*Xenostigminazilleri*	CBS 115685	GU269840	GU253857	LC203316	GU384553

Abbreviations: ANM, A.N. Miller; AFTOL, Assembling the Fungal Tree of Life project; CBS, Centraalbureau voor Schimmelcultures, Utrecht, The Netherlands; CPC, Working collection of Pedro Crous housed at CBS; DAOM, Plant Research Institute, Department of Agriculture (Mycology), Ottawa, Canada; GLMC, culture collections of the Senckenberg Museum of Natural History Görlitz, Germany; GKM, G.K. Mugambi; IL, I. Lopez; KUMCC, Culture Collection of Chinese Academy of Sciences, Kunming, China; KH, K. Hirayama; KT, K. Tanaka; MFLU, Mae Fah Luang University; MFLUCC, Mae Fah Luang University Culture Collection; NCYUCC, National Chiayi University Culture Collection, China; SMH, S.M. Huhndorf; ZHKUCC, Zhongkai University of Agriculture and Engineering Culture Collection, China.

### ﻿Phylogenetic analyses

The obtained sequences were initially assembled (forward and reverse sequences) using SeqMan v. 7.0.0 (DNASTAR, Madison, WI). Assembled sequences were subjected to BLAST search in GenBank to obtain strains which have high similarities (https://blast.ncbi.nlm.nih.gov/). In addition, some other sequences for taxa in Melanommataceae were obtained using recent publications (Kularathnage et al. 2022; Gao et al. 2023). In total, 85 isolates were used for phylogenetic analyses including *Hysteriumangustatum* (MFLU 16-1179) as the outgroup taxon. The combined dataset comprised four genes, ITS, LSU, SSU and *tef1-α*. Single gene sequences were aligned with MAFFT v.7.490 online application (Katoh and Standley 2013) (https://mafft.cbrc.jp/alignment/software/) and manually improved in necessary places. Aligned sequences were combined using BioEdit v.7.2.5 (Hall 1999).

The concatenated aligned dataset was analysed separately using Maximum Likelihood (ML) and Bayesian Inference (BI). Maximum Likelihood analysis was performed using the online portal CIPRES Science Gateway v. 3.3 (Miller et al. 2010), with RAxML-HPC v.8 on XSEDE (8.2.12) tool (Stamatakis et al. 2008; Stamatakis 2014) using the GTR+I+G model of nucleotide evolution. Evolutionary models for each barcode were determined using MrModelTest v. 2.3 (Nylander 2004) under the Akaike Information Criterion (AIC).

MrBayes 3.2.1 (Ronquist et al. 2012) was used to analyse Bayesian Inference phylogenies and was run with four chains of 3,000,000 generations and trees were sampled every 100^th^ generation. The initial 20% of sampled data were discarded as burn-in. The phylograms were visualised using the FigTree v.1.4.0 tool (Rambaut 2012) and reorganised in Adobe Illustrator® CS3 (Version 15.0.0, Adobe®, San Jose, CA). The alignments and sequences were deposited in TreeBASE, submission ID: 31212 (http://www.treebase.org/) and GenBank (https://www.ncbi.nlm.nih.gov/), respectively.

### ﻿Geographical distribution and host associations of melanommataceous species

We enumerated 340 species of Melanommataceae, grouped into 36 genera, along with their geographic distribution and host associations. The necessary data were obtained from published books, publications in reputable journals, Species Fungorum (https://www.speciesfungorum.org), MycoBank (https://www.mycobank.org/), the U.S. National Fungus Collections Fungus-Host Database (Farr and Rossman 2024) and online sources (Catalogue of Life Checklist). The gathered data were mentioned with appropriate references (Table 2). In total, 296 articles were studied for information on the 340 melanommataceous species that are currently legitimate (Table 2) and accessed through Google Scholar searches. MycoBank (https://www.mycobank.org/) was used to illustrate the nomenclature validity of the taxa. However, numerous species have not been verified using molecular data, so most species should be correctly identified, based on modern taxonomic concepts. Therefore, the distribution may vary slightly.

**Table 2.** Host association and geographical distribution of reported melanommataceous species.

Species	Host	Host family	Locality	References
*Alpinariarhododendri*	*Rhododendron* sp.	Ericaceae	Austria and Japan	Hashimoto et al. (2017); Jaklitsch and Voglmayr (2017)
*Aposphaeriaallantella*	*Solanumtuberosum*	Solanaceae	Germany	Farr and Rossman (2024)
*Aposphaeriaanomala*	Unidentified host	–	Italy	Farr and Rossman (2024)
*Aposphaeriaarachidis*	*Arachishypogaea*	Fabaceae	India	Kulkarni (1974); Mathur (1979); Farr and Rossman (2024)
*Aposphaeriabambusae*	*Bambusa* sp.	Poaceae	Brazil	Bresadola (1926); Farr and Rossman (2024)
*Aposphaeriabombacis*	*Bombaxmacrocarpum*	Malvaceae	Germany	Diedicke (1912)
*Aposphaeriabrunneotincta*	*Castaneavesca*	Fagaceae	The United States	Thaxter (1922)
*Aposphaeriabuddlejae*	*Buddlejadavidii*	Buddlejaceae	Ukraine	Farr and Rossman (2024)
*Aposphaeriacalligoni*	*Calligonumaphyllum*	Polygonaceae	Kazakhstan	Farr and Rossman (2024)
*Aposphaeriacanavaliae*	*Canavalia* sp.	Fabaceae	Fiji	Massee (1906); Dingley et al. (1981); Farr and Rossman (2024)
*Aposphaeriacaraganae*	*Caraganaarborescens*	Fabaceae	Russia	Farr and Rossman (2024)
*Aposphaeriacaricicola*	*Carexrossii*	Cyperaceae	The United States	Farr and Rossman (2024)
*Aposphaeriacaulina*	*Cerefoliumsylvestre*	Apiaceae	Finland	Karsten (1905)
*Aposphaeriacharticola*	–	–	The United States	Saccardo (1911)
*Aposphaeriacladoniae*	*Cladoniafimbriata*	Cladoniaceae	Germany	Allescher (1896)
*Aposphaeriaconica*	*Quercus* sp.	Fagaceae	Italy	Saccardo and Traverso (1910)
*Aposphaeriacorallinolutea*	*Fraxinusexcelsior* and *Kerriajaponica*	Oleaceae and Rosaceae	The Netherlands	De Gruyter et al. (2013)
*Aposphaeriadendrophomoides*	*Corylusavellana*	Betulaceae	Italy	Saccardo (1921)
*Aposphaeriadenudata*	*Cydoniavulgaris*	Rosaceae	Hungary	Saccardo and Traverso (1910)
*Aposphaeriadesertorum*	*Haloxylonaphyllum*	Amaranthaceae	Kazakhstan	Farr and Rossman (2024)
*Aposphaeriaelymi*	*Elymusarenarius*	Poaceae	Germany	Diedicke (1912)
*Aposphaeriaephedrae*	*Ephedra* sp.	Ephedraceae	Kazakhstan and Ukraine	Hayova and Minter (2009); Farr and Rossman (2024)
*Aposphaeriaepicorticalis*	*Corylusavellana*	Betulaceae	Italy	Saccardo (1921)
*Aposphaeriaeragrostidis*	*Eragrostis* sp.	Poaceae	Eritrea, Ethiopia and Iraq	Castellani and Ciferri (1950); Farr and Rossman (2024)
*Aposphaeriaeurotiae*	*Eurotiaeversmanniana*	Amaranthaceae	Kazakhstan	Farr and Rossman (2024)
*Aposphaeriaferrum-equinum*	Unidentified host	–	Italy	Tassi (1900)
*Aposphaeriafreticola*	*Fagus* sp. and *Nothofagusantarctica*	Fagaceae, Nothofagaceae	Argentina, Chile and Poland	Farr (1973); Mulenko et al. (2008); Farr and Rossman (2024)
*Aposphaeriagallicola*	Unidentified host	–	Italy	Farr and Rossman (2024)
*Aposphaeriagregaria*	*Salix* sp.	Salicaceae	Germany	Diedicke (1912)
*Aposphaeriahalimodendri*	*Halimodendronhalodendron*	Fabaceae	Ukraine	Farr and Rossman (2024)
*Aposphaeriahaloxyli*	*Haloxylonaphyllum*	Amaranthaceae	Kazakhstan	Farr and Rossman (2024)
*Aposphaeriahapalophragmii*	*Hapalophragmiumacaciae*	Fabaceae	Somalia	Trotter (1931); Mujica and Vergara (1945); Farr and Rossman (2024)
*Aposphaeriahenryana*	*Salixalba*	Salicaceae	Italy	Farr and Rossman (2024)
*Aposphaeriaheveae*	*Heveabrasiliensis*	Euphorbiaceae	Sri Lanka	Petch (1917)
*Aposphaeriahippuridis*	*Hippurisvulgaris*	Plantaginaceae	Germany	Ade (1923)
*Aposphaeriahospitae*	*Kleinhovia* sp.	Malvaceae	Sri Lanka	Tassi (1900)
*Aposphaeriahumicola*	Unidentified host	–	The Netherlands	Oudemans (1902)
*Aposphaeriailicis*	*Ilexaquifolium*	Aquifoliaceae	Germany	Diedicke (1912)
*Aposphaeriailiensis*	*Halimodendronhalodendron*	Fabaceae	Kazakhstan	Farr and Rossman (2024)
*Aposphaeriajubaeae*	*Jubaeaspectabilis*	Arecaceae	Chile	Spegazzini (1921); Farr (1973); Mujica and Vergara (1945); Farr and Rossman (2024)
*Aposphaeriakiefferiana*	*Quercus* sp.	Fagaceae	Italy	Farr and Rossman (2024)
*Aposphaeriakravtzevii*	*Eurotiaeversmanniana*	Amaranthaceae	Kazakhstan	Farr and Rossman (2024)
*Aposphaerialentisci*	*Pistacia* sp.	Anacardiaceae	Greece and Spain	Urries (1957); Pantidou (1973); Farr and Rossman (2024)
*Aposphaerialignicola*	*Acaciaarabica*	Fabaceae	Pakistan	Ahmad (1964)
*Aposphaeriamajor*	*Rubusparviflorus*	Rosaceae	The United States	Sydow and Sydow (1907)
*Aposphaeriamajuscula*	*Vitisvinifera*	Vitaceae	France	Saccardo and Trotter (1913)
*Aposphaeriamartinii*	*Sabal* sp.	Arecaceae	United States	Farr and Rossman (2024)
*Aposphaeriamediella*	*Pinus* sp.	Pinaceae	Greece, Finland and Poland	Karsten (1884); Pantidou (1973); Mulenko et al. (2008); Farr and Rossman (2024)
*Aposphaeriamelaleucae*	*Melaleucaleucadendra*	Myrtaceae	Australia	Hennings (1903); Shivas and Alcorn (1996); Farr and Rossman (2024)
*Aposphaeriamesembryanthemi*	*Mesembryanthemum* sp.	Aizoaceae	Portugal	Costa and Camara (1952)
*Aposphaeriamojunkumica*	*Haloxylonaphyllum*	Amaranthaceae	Kazakhstan	Farr and Rossman (2024)
*Aposphaeriamontbretiae*	*Crocosmia* sp.	Iridaceae	Azerbaijan and Georgia	Siemaszko (1923)
*Aposphaeriamusarum*	*Musasapientum*	Musaceae	Argentina	Farr (1973); Farr and Rossman (2024)
*Aposphaerianigra*	*Betulaalba*	Betulaceae	Germany	Diedicke (1912)
*Aposphaeriaoxalidis*	*Oxalistuberosa*	Oxalidaceae	Bolivia	Farr and Stevenson (1963); Farr and Rossman (2024)
*Aposphaeriapakistanica*	Unidentified host	–	Pakistan	Ahmad (1971)
*Aposphaeriaphellodendri*	*Phellodendronamurense*	Rutaceae	Ukraine	Farr and Rossman (2024)
*Aposphaeriapinea*	*Pinussylvestris*	Pinaceae	France and Germany	Saccardo (1884); Mulenko et al. (2008); Farr and Rossman (2024)
*Aposphaeriapini-densiflorae*	*Pinusdensiflora*	Pinaceae	Japan	Sawada (1950); Farr and Rossman (2024)
*Aposphaeriapolonica*	*Tiliaplatyphyllos*	Pinaceae	Poland	Mulenko et al. (2008); Farr and Rossman (2024)
*Aposphaeriapopulea*	*Populus* sp.	Salicaceae	The United Kingdom	Smith and Ramsbottom (1914); Dennis (1986)
*Aposphaeriapulviscula*	*Fagussylvatica* and *Salix* sp.	Fagaceae and Salicaceae	Austria, France, Iceland, Italy, the Netherlands and Ukraine	Saccardo (1880); Spaulding (1961); Sutton (1980); Farr and Rossman (2024)
*Aposphaeriapunicina*	*Punicagranatum*	Lythraceae	China and Malta	Saccardo (1915); Tai (1979)
*Aposphaeriapurpurascens*	*Acerpseudoplatanus*	Sapindaceae	Italy	Peyronel (1915)
*Aposphaeriaramalinae*	*Ramalina* sp. (lichen)	–	France	Pitard and Harmand (1911)
*Aposphaeriareaumuriae*	*Reaumuria* sp.	Tamaricaceae	Azerbaijan	Farr and Rossman (2024)
*Aposphaeriarhois*	*Rhusoxyacantha*	Anacardiaceae	Libya	El-Buni and Rattan (1981); Trotter (1912)
*Aposphaeriarostrata*	Unidentified host	–	The Netherlands	Oudemans (1902)
*Aposphaeriarubefaciens*	*Salix* sp.	Salicaceae	Italy and the Netherlands	Bubák and Kabát (1905)
*Aposphaeriarudis*	*Piceaexcelsa*	Pinaceae	Finland	Karsten (1905)
*Aposphaeriasalicis*	*Salix* sp.	Salicaceae	Germany and India	Diedicke (1912); Mathur (1979)
*Aposphaeriasalicum*	*Salixviminalis*	Salicaceae	Germany	Sydow and Sydow (1903)
*Aposphaeriasantolinae*	*Santolinachamaecyparissus*	Asteraceae	Ukraine	Farr and Rossman (2024)
*Aposphaeriasepulta*	*Citrusaurantium*	Rutaceae	Italy	Saccardo (1884)
*Aposphaeriasequoiae*	*Sequoia* sp.	Cupressaceae	Denmark	Lind (1913)
*Aposphaeriasilenes*	*Sileneotites*	Caryophyllaceae	Russia	Farr and Rossman (2024)
*Aposphaeriasphaerospora*	*Betulaalba*	Betulaceae	Italy	Peyronel (1918)
*Aposphaeriastriolata*	*Populusdeltoides*	Salicaceae	The United States	Saccardo (1916)
*Aposphaeriataquarae*	Bambusoideae sp.	Poaceae	Brazil	Viégas (1945)
*Aposphaeriatiliana*	*Tiliacordata*	Malvaceae	Ukraine	Gucevic (1977)
*Aposphaeriatragopogonis*	*Tragopogondubius*	Asteraceae	Romania	Sandu-Ville and Mititiuc (1971)
*Aposphaeriaturmalis*	*Diospyrosvirginiana*	Ebenaceae	The United States	Ellis and Everhart (1902); Cash (1952)
*Aposphaeriaulmicola*	*Ulmus* sp.	Ulmaceae	The United Kingdom	Saccardo (1884)
*Aposphaeriazeae*	*Zeamays*	Poaceae	Azerbaijan and Georgia	Farr and Rossman (2024)
*Asymmetricosporacalamicola*	*Calamuscaryotoides*	Arecaceae	Australia	Fröhlich and Hyde (1998); Zhang et al. (2012)
*Bertiellabotryosa*	*Ulmus* sp.	Ulmaceae	The United States	Morgan (1904)
*Bertiellaellipsoidea*	Unidentified host	–	Thailand	Hyde et al. (2016)
*Bertiellafici*	*Cinnamomumverum*, *Ficusseptica*	Lauraceae, Moraceae	China and Thailand	Tennakoon et al. (2021); this study
*Bertiellagelatinosa*	Unidentified host	–	Brazil	Almeida et al. (2017)
*Bertiellarhodospila*	*Cyrilla* sp., *Populus* sp. and *Quercus* sp.	Cyrillaceae, Fagaceae and Salicaea	The United States	Barr et al. (1986)
*Bertiellastriatispora*	Unidentified host	–	India	Niranjan and Sarma (2019)
*Bicrouaniamaritima*	*Atraphaxisspinosa* and *Halimioneportulacoides*	Amaranthaceae, Polygonaceae	France and Uzbekistan	Kohlmeyer and Volkmann-Kohlmeyer (1990); Gafforov (2017)
*Byssosphaeriaalnea*	*Alnus* sp.	Betulaceae	The United States	Barr (1984); Farr and Rossman (2024)
*Byssosphaeriaerumpens*	*Litsea* sp.	Lauraceae	China	Chen and Hsieh (2004); Li and Zhuang (2008); Farr and Rossman (2024)
*Byssosphaeriaerythrinae*	*Erythrinaindica*	Fabaceae	France	Barr (1984)
*Byssosphaeriaguangdongense*	*Phoenixroebelenii*	Arecaceae	China	Xiong et al. (2023)
*Byssosphaeriahainanensis*	Unidentified host	–	China	Li and Zhuang (2008)
*Byssosphaeriajamaicana*	*Bambusa* sp., *Quercus* sp. and *Salix* sp.	Fagaceae and Poaceae, Salicaceae	China, Czech Republic, Jamaica and Mexico	Barr (1984); Sivanesan and Hsieh (1989); Wang et al. (2004); Medel (2007); Farr and Rossman (2024)
*Byssosphaeriajuniperi*	*Juniperus* sp.	Cupressaceae	The United States	Wang et al. (2004); Farr and Rossman (2024)
*Byssosphaeriamacarangae*	*Macarangatanarius*	Euphorbiaceae	China	Tennakoon et al. (2018)
*Byssosphaeriamusae*	*Musa* sp.	Musaceae	Thailand	Liu et al. (2015); Farr and Rossman (2024)
*Byssosphaeriaoviformis*	*Saccharumarundinaceum*	Poaceae	China and Jamaica	Barr (1984); Lu et al. (2000); Wong and Hyde (2001); Farr and Rossman (2024)
*Byssosphaeriaphoenicis*	*Phoenixroebelenii*	Arecaceae	China	Kularathnage et al. (2022)
*Byssosphaeriapoaceicola*	*Arundopliniana*	Poaceae	China	This study
*Byssosphaeriarhodomphala*	*Acerpseudoplatanus* and *Populus* sp.	Salicaceae and Sapindaceae	Brazil, China, Poland and the United States	Cooke (1887); Scheuer and Chlebicki (1997); Reblova (1997); Chen and Hsieh (2004); Wang et al. (2004)
*Byssosphaeriasalebrosa*	*Vaccinium* sp.	Ericaceae	The United States	Barr (1984); Barr et al. (1986); Farr and Rossman (2024)
*Byssosphaeriaschiedermayriana*	*Sambucusnigra*	Adoxaceae	Austria	Barr (1984)
*Byssosphaeriasemen*	*Pyrusamericana*	Rosaceae	The United States	Barr (1984)
*Byssosphaeriasiamensis*	*Pandanus* sp. and *Citrustrifoliata*	Pandanaceae and Rutaceae	China and Thailand	Tian et al. (2015); Hyde et al. (2018); Farr and Rossman (2024); this study
*Byssosphaeriataiwanense*	*Macarangatanarius*	Euphorbiaceae	China	Tennakoon et al. (2018)
*Byssosphaeriaxestothele*	*Cornusflorida* and *Robiniapseudoacacia*	Cornaceae and Fabaceae	Sweden and the United States	Barr (1984); Eriksson (2014); Farr and Rossman (2024)
*Calyptronectriaargentinensis*	*Foeniculumpiperitum* and *Manihotcarthaginensis*	Apiaceae, Euphorbiaceae	Argentina	Spegazzini (1909); Farr (1973); Farr and Rossman (2024)
*Calyptronectriaindica*	*Annonasquamosa*	Annonaceae	India	Pande (2008)
*Calyptronectriaplatensis*	*Manihotcarthagenensis*	Euphorbiaceae	Argentina	Spegazzini (1909); Farr (1973); Farr and Rossman (2024)
*Camposporiumantennatum*	*Acaciaaulacocarpa*, *Caesalpiniaechinata*, *Cinnamomumjaponicum*, *Cocosnucifera*, *Drymophloeuspachycladus*, *Eucalyptusglobulus*, *Ficuserecta*, *Laurusnobilis*, *Machilus* sp., *Mucunaferruginea*, *Neolitseascrobiculata*, *Phoenixhanceana*, *Pinusmassoniana*, *Quercus* sp. and *Trachycarpusfortunei*	Arecaceae, Fabaceae, Fagaceae, Lauraceae, Moraceae, Myrtaceae, and Pinaceae	California, China, the United Kingdom and Venezuela	Harkness (1884); Lu et al. (2000); Castaneda-Ruiz et al. (2003); Farr and Rossman (2024)
*Camposporiumappendiculatum*	Unidentified host	–	China	Hyde et al. (2020)
*Camposporiumatypicum*	*Mesuaferrea*	Calophyllaceae	India	Koukol and Delgado (2021)
*Camposporiumcambrense*	*Alnus* sp., *Carpinusbetulus*, *Fagus* sp., *Freycinetiaanksia*, *Laurusnobilis* and *Quercus* sp.	Betulaceae, Fagaceae, Lauraceae, and Pandanaceae	China, New Zealand, Poland, Russia and the United Kingdom	Hughes (1951); Whitton et al. (2002); Mulenko et al. (2008); Farr and Rossman (2024)
*Camposporiumchinense*	Unidentified host	–	China	Xu et al. (2021)
*Camposporiumdulciaquae*	Unidentified host	–	Thailand	Calabon et al. (2021)
*Camposporiumfusisporum*	*Pandanus* sp.	Pandanaceae	Brunei	Whitton et al. (2002)
*Camposporiumhimalayanum*	*Phoenix* sp.	Arecaceae	India	Adamcik et al. (2015)
*Camposporiumhyalinum*	*Elaeagnusmacrophylla*, and *Fagus* sp.	Elaeagnaceae and Fagaceae	The United Kingdom	Abdullah (1980); Kirk and Spooner (1984)
*Camposporiumhyderabadense*	*Borassusflabellifer*, *Machilusthunbergii* and *Mucunaferruginea*	Arecaceae, Fabaceae, and Lauraceae	China, India and Japan	Rao and Rao (1964); Ichinoe (1971); Farr and Rossman (2024)
*Camposporiumindicum*	*Borassusflabellifer*	Arecaceae	India	Rao and Rao (1964)
*Camposporiumjaponicum*	*Acaciaconfusa*, *Betulapendula*, *Cinnamomumjaponicum*, *Freycinetiaarborea*, *Litchichinensis*, *Machilusthunbergii*, *Mucunaferruginea*, *Paulowniakawakamii* and *Quercus* sp.	Betulaceae, Lauraceae, Pandanaceae, Paulowniaceae, and Sapindaceae	China and Japan	Ichinoe (1971); Matsushima (1980); Farr and Rossman (2024)
*Camposporiumlaundonii*	*Araliaelata*, *Pasaniaedulis* and *Rosa* sp.	Araliaceae, Fagaceae, and Rosaceae	Japan and New Zealand	Ellis (1976); Watanabe (1993); Farr and Rossman (2024)
*Camposporiumlycopodiellae*	*Lycopodiellainundata*	Lycopodiaceae	Germany	Hyde et al. (2020)
*Camposporiummarylandicum*	Unidentified host	–	The United States	Shearer (1974)
*Camposporiummicrosporum*	*Borassusflabellifer*	Arecaceae	India	Rao and Rao (1964)
*Camposporiummultiseptatum*	Unidentified host	–	China	Hyde et al. (2020)
*Camposporiumontariense*	*Acersaccharum* and *Freycinetiaarborea*	Sapindaceae and Pandanaceae	Canada and the United States	Matsushima (1983); Whitton et al. (2002)
*Camposporiumpellucidum*	*Betulapendula*, *Caesalpiniaechinata*, *Carpodetusserratus*, *Elaeagnus* sp., *Fagus* sp., *Laurus* sp., *Pasaniaglabra*, *Piceaabies*, *Rhopalostylis* sp. and *Sorbusaucuparia*	Areceae, Betulaceae, Elaeagnaceae, Fabaceae, Lauraceae, Pinaceae, Rosaceae, and Rousseaceae	Brazil, Japan, Poland, New Zealand, Russia and the United Kingdom	Hughes (1951); Matsushima (1983); Mulenko et al. (2008); Farr and Rossman (2024)
*Camposporiumquercicola*	*Quercusgermana*	Fagaceae	Mexico	Mercado Sierra et al. (1995)
*Camposporiumramosum*	*Freycinetia* sp.		Australia and the United States	Whitton et al. (2002)
*Camposporiumscolecosporium*	Unidentified host	–	Papua New Guinea	Kobayasi (1971)
*Camposporiumseptatum*	Unidentified host	–	Thailand	Hyde et al. (2020)
*Camposporiumvaldivianum*	*Sophoramicrophylla*	Fabaceae	The United States	Koukol and Delgado (2021)
*Camposporiumverruculosum*	*Clematisvitalba*	Ranunculaceae	Italy	Koukol and Delgado (2021)
*Dematiomelanommayunnanense*	*Hypericummonogynum* and *Rubusparvifolius*	Hypericaceae and Rosaceae	China	Gao et al. (2023)
*Exosporiellafungorum*	*Thelephora* sp. (leathery earthfans/mushroom sp.)	–	Sweden	Karsten (1892)
*Fusiconidiummackenziei*	*Clematisvitalba*	Ranunculaceae	Italy	Li et al. (2017)
*Herpotrichiaalligata*	*Opuntia* sp.	Cactaceae	Sweden and the United States	Barr (1992); Farr and Rossman (2024)
*Herpotrichiaalpincola*	*Aconitum* sp.	Ranunculaceae	Hungary and Slovakia	Rehm (1906)
*Herpotrichiaarizonica*	*Carnegieagigantea*	Cactaceae	The United States	Barr (1992); Farr and Rossman (2024)
*Herpotrichiaaustralis*	*Sclerocaryacaffra*	Anacardiaceae	South Africa	Bose (1961)
*Herpotrichiabakeri*	*Sambucusjavanica*	Viburnaceae	Philippines	Teodoro (1937); Farr and Rossman (2024)
*Herpotrichiabambusana*	*Bambusavulgaris*	Poaceae	Brazil	Hennings (1908); Eriksson and Yue (1998)
*Herpotrichiaboldoae*	*Boldeafragrans* and *Peumusboldus*	Monimiaceae	Chile	Spegazzini (1910); Mujica and Vergara (1945); Spaulding (1961); Farr (1973); Farr and Rossman (2024)
*Herpotrichiabrasiliensis*	Unidentified host	–	Brazil	Rick (1933)
*Herpotrichiabrenckleana*	*Urticagracilis*	Urticaceae	Sweden	Eriksson (2014); Farr and Rossman (2024
*Herpotrichiacaesalpiniae*	*Caesalpiniasepiaria*	Fabaceae	South Africa	Doidge (1948); Sivanesan (1971)
*Herpotrichiacalamicola*	*Calamuscaryotoides*	Arecaceae	Australia	Fröhlich and Hyde (2000); Farr and Rossman (2024)
*Herpotrichiacallimorpha*	*Chamaenerionangustifolium*, *Salix* sp. and *Xanthophyllumflavescens*	Onagraceae, Polygalaceae, and Salicaceae	Denmark and India	Munk (1957); Rabenhorst (1869); Pande (2008); Farr and Rossman (2024)
*Herpotrichiacaulogena*	*Silenenutans*	Caryophyllaceae	Luxembourg	Feltgen (1903)
*Herpotrichiachilensis*	*Proustiapungens*	Asteraceae	Chile	Feltgen (1903); Farr (1973); Farr and Rossman (2024)
*Herpotrichiacirrhostoma*	Unidentified host	–	Sri Lanka	Berkeley and Broome (1873); Petch (1912)
*Herpotrichiadalisayi*	Unidentified host	–	The Philippines	Hyde and Aptroot (1998)
*Herpotrichiadecidua*	Unidentified host	–	The United States	Barr (1992); Farr and Rossman (2024)
*Herpotrichiadetzneriae*	*Detzneriatubata*	Plantaginaceae	Papua New Guinea	Kobayasi (1971); Shaw (1984); Farr and Rossman (2024)
*Herpotrichiadiffusa*	*Juglanscinerea* and *Populus* sp.	Juglandaceae	Palestine and the United States	Ellis and Everhart (1892); Ellis (1895)
*Herpotrichiaellisii*	*Abies* sp.	Pinaceae	Canada	Barr (1992); Farr and Rossman (2024)
*Herpotrichiaephedrae*	*Ephedradistachya*	Ephedraceae	France	Kuhnholtz-Lordat and Barry (1949)
*Herpotrichiafusispora*	Unidentified host	–	China	Chen and Hsieh (2004); Farr and Rossman (2024)
*Herpotrichiagelasinosporoides*	Soil	–	India	Von Arx (1981)
*Herpotrichiahenkeliana*	*Phragmitescommunis*	Poaceae	Germany	Sydow and Sydow (1921)
*Herpotrichiaherbarum*	*Achilleamillefolium*	Asteraceae		Wehmeyer (1952); Barr (1992)
*Herpotrichiaherpotrichoides*	*Carya* sp., *Epilobium* sp., *Rhododendronhirsutum*, *Ribes* sp. and *Rubus* sp.	Ericaceae, Juglandaceae, Grossulariaceae, Onagraceae, and Rosaceae	Austria, Denmark, Germany, Poland, the United States and the United Kingdom	Cannon (1982); Barr (1984); Cannon et al. (1985); Mulenko et al. (2008); Zhang et al. (2012); Tian et al. (2015); Farr and Rossman (2024)
*Herpotrichiahippocrateae*	*Hippocrateagrahamii*	Celastraceae	India	Tilak and Talde (1975); Pande (2008)
*Herpotrichiaindica*	*Durantaplumieri*	Verbenaceae	India	Anahosur (1970); Pande (2008); Farr and Rossman (2024)
*Herpotrichialaricina*	*Larixdecidua*	Pinaceae	Luxembourg	Feltgen (1901)
*Herpotrichialeptospora*	Unidentified host	–	–	Kirschstein (1911)
*Herpotrichialignicola*	Unidentified host	–	Belgium	Bose (1961)
*Herpotrichiamacrotricha*	*Acerspicatum*, *Agropyronrepens*, *Agrostisalba*, *Arundinaria* sp., *Carex* sp., *Cocosnucifera*, *Eupatoriumformosanum*, *Fagussylvatica*, *Fraxinus* sp., *Rubus* sp. and *Solidago* sp.	Arecaceae, Asteraceae, Cupressaceae, Cyperaceae, Fagaceae, Oleaceae, Poaceae, Rosaceae, and Sapindaceae	China, India, the United Kingdom and the United States	Dennis (1978); Saccardo (1883); Barr (1968, 1984); Pande (2008); Chen and Hsieh (2004); Farr and Rossman (2024)
*Herpotrichiamangrovei*	Unidentified host	–	China	Jones and Vrijmoed (2003)
*Herpotrichiamelanotricha*	*Heveabrasiliensis*	Euphorbiaceae	Congo	Saccas (1954)
*Herpotrichiamillettiae*	*Millettia* sp.	Fabaceae	Malaysia	Sivanesan (1971)
*Herpotrichiamonospermatis*	*Buteamonosperma*	Fabaceae	India	Pande (2008); Farr and Rossman (2024)
*Herpotrichiamulleri*	*Artemisianilagirica* and *Buteamonosperma*	Asteraceae, Fabaceae	India	Pande (2008); Farr and Rossman (2024)
*Herpotrichiamyriangii*	*Caricapapaya*	Caricaceae	Java	Raciborski (1909)
*Herpotrichianectrioides*	Melastomataceae sp.	Melastomataceae	Brazil	Rehm (1901)
*Herpotrichianigra*	*Abies* sp., *Calocedrusdecurrens*, *Cedruslibani*, *Chamaecyparisnootkatensis*, *Juniperus* sp., *Phyllodoce* sp., *Picea* sp., *Pinus* sp., *Pseudotsugamenziesii* and *Rhododendron* sp.	Cupressaceae, Ericaceae, and Pinaceae	Austria, Canada, France, Germany, Italy, Norway, Poland, Switzerland, Turkey, Ukraine and the United States	Shaw (1973); Ginns (1986); French (1989); Farr and Rossman (2024)
*Herpotrichianigrotuberculata*	*Elaeisguineensis* and *Phyllostachysreticulata*	Arecaceae and Poaceae	Japan and Tanzania	Pirozynski (1972); Farr and Rossman (2024)
*Herpotrichianypicola*	*Nypafruticans*	Arecaceae	Malaysia	Hyde et al. (1999); Farr and Rossman (2024)
*Herpotrichiaocculta*	*Eucalyptus* sp.	Myrtaceae	Brazil	Rick (1933)
*Herpotrichiaochrostoma*	*Fraxinusexcelsior*	Oleaceae	Luxembourg	Feltgen (1903)
*Herpotrichiapalmicola*	*Calamuscaryotoides*, *Daemonorops* sp. and *Licualaramsayi*	Arecaceae	Australia and China	Hyde et al. (1999); Fröhlich and Hyde 2000; Zhuang (2001); Farr and Rossman (2024)
*Herpotrichiapandei*	*Saccharumspontaneum*	Poaceae	India	Bose (1961)
*Herpotrichiapetrakiana*	*Fagussylvatica*	Fagaceae	–	Bose (1961)
*Herpotrichiaphilippinensis*	*Alstoniascholaris*	Apocynaceae	The Philippines	Rehm (1914); Teodoro (1937); Farr and Rossman (2024)
*Herpotrichiapinetorum*	*Herpotrichiapinetorum*	Pinaceae	Austria and India	Winter (1885); Mueller (1958); Petrak (1962); Farr and Rossman (2024)
*Herpotrichiaquinqueseptata*	*Abieslasiocarpa*, *Larixeuropaea*, *Picea* sp. and *Populustremula*	Pinaceae and Salicaceae	Canada, Czech Republic, Germany, Sweden and the United States	Shaw (1973); Minter (1981); Ginns (1986); Eriksson (2014); Farr and Rossman (2024)
*Herpotrichiarara*	*Tanacetumvulgare*	Asteraceae	Germany	Kirschstein (1935)
*Herpotrichiarhenana*	*Epilobiumangustifolium*, *Rhododendronhirsutum* and *Ribes* sp.	Ericaceae, Grossulariaceae, and Onagraceae	Austria and the United States	Fuckel (1870); Remler (1979); Farr and Rossman (2024)
*Herpotrichiarhodospiloides*	*Populusdeltoides*	Salicaceae	The United States	Peck (1909)
*Herpotrichiarhodosticta*	*Carexpaniculata* and *Populus* sp.	Cyperaceae and Salicaceae	Germany and the United States	Saccardo (1882); Samuels (1976); Farr and Rossman (2024)
*Herpotrichiasetosa*	*Betulaglandulosa* and *Myricagale*	Betulaceae and Myricaceae	Canada and Ireland	Barr (1992); Farr and Rossman (2024)
*Herpotrichiastriatispora*	Soil	–	South Africa	Papendorf and Arx (1966)
*Herpotrichiasymphoricarpi*	*Symphoricarpos* sp.	Caprifoliaceae	The United States	Barr (1984); Farr and Rossman (2024)
*Herpotrichiatenuispora*	*Urticadioica*	Urticaceae	Germany	Kirschstein (1906)
*Herpotrichiavaginatispora*	*Trifolium* sp.	Fabaceae	Italy	Tian et al. (2015)
*Herpotrichiavillosa*	Unidentified host	–	Brazil	Samuels and Müller (1978)
*Herpotrichiaxiaokongensis*	*Prunus* sp.	Rosaceae	China	Hyde et al. (2021)
*Herpotrichiazingiberacearum*	*Hedychiumcoronarium*	Zingiberaceae	China	This study
*Mamillisphaeriadimorphospora*	Unidentified host	–	Australia	Hyde et al. (1996)
*Marjiatianshanica*	*Cerasustianshanica*	Rosaceae	Uzbekistan	Wanasinghe et al. (2018)
*Marjiauzbekistanica*	*Rosa* sp.	Rosaceae	Uzbekistan	Wanasinghe et al. (2018)
*Melanocamarosporioidesugamica*	*Loniceraaltmannii*	Caprifoliaceae	Uzbekistan	Pem et al. (2019)
*Melanocamarosporiumgaliicola*	*Galium* sp.	Rubiaceae	Italy	Wijayawardene et al. (2016)
*Melanocucurbitariauzbekistanica*	*Acerpubescens*	Sapindaceae	Uzbekistan	Wanasinghe et al. (2018)
*Melanodiplodiatianschanica*	*Rosa* sp.	Rosaceae	Uzbekistan	Wanasinghe et al. (2018)
*Melanommaacanthophilum*	*Cereusquisco*	Cactaceae	Chile	Spegazzini (1923); Farr (1973); Farr and Rossman (2024)
*Melanommaafflatum*	Unidentified host	–	The United States	Shear (1941)
*Melanommaanceps*	Unidentified host	–	Jawa	Höhnel (1909)
*Melanommaandinum*	*Bulnesiaretamo*	Zygophyllaceae	Argentina	Spegazzini (1909); Farr (1973); Farr and Rossman (2024)
*Melanommaartemisiae-maritimae*	*Artemisiamaritima*	Asteraceae	Russia	Lobik (1927); Farr and Rossman (2024)
*Melanommaaspegrenii*	*Carpinusbetulus*, *Cornus* sp. and *Fagussylvatica*	Betulaceae, Cornaceae, and Fagaceae	Poland	Fuckel (1870); Mulenko et al. (2008); Farr and Rossman (2024)
*Melanommaaurantiicola*	*Citrus* sp.	Rutaceae	Paraguay	Spegazzini (1920); Farr (1973); Farr and Rossman (2024)
*Melanommaaurantiiphila*	*Citrus* sp.	Rutaceae	Paraguay	Spegazzini (1920); Farr (1973); Farr and Rossman (2024)
*Melanommaaustraliense*	Unidentified host	–	Australia	Hyde and Goh (1999)
*Melanommabrachythele*	*Sambucus* sp.	Adoxaceae	The United Kingdom	Saccardo (1878)
*Melanommabubakii*	*Campanulastricta*	Campanulaceae	Turkey	Bubák (1914)
*Melanommacacheutense*	*Baccharisglutinosa*	Asteraceae	Argentina	Spegazzini (1909); Farr (1973); Farr and Rossman (2024)
*Melanommacaesalpiniae*	*Caesalpiniacearense*	Fabaceae	Brazil	Hennings (1908)
*Melanommacaryophagum*	*Carya* sp. and *Juglans* sp.	Juglandaceae	The United States	Fairman (1921)
*Melanommacastillejae*	*Castillejapallida*	Orobanchaceae	Siberia	Farr and Rossman (2024)
*Melanommaceratoniae*	*Ceratoniasiliqua*	Fabaceae	Spain	Farr and Rossman (2024)
*Melanommachilense*	*Proustiapungens*	Asteraceae	Chile	Spegazzini (1910); Farr (1973); Farr and Rossman (2024)
*Melanommacitricola*	*Citrusmedica*	Rutaceae	Bangladesh and India	Sydow et al. (1911); Rao (1969); Farr and Rossman (2024)
*Melanommaconjunctum*	*Thujaplicata*	Cupressaceae	The United States	Farr and Rossman (2024)
*Melanommacryptostegiae*	*Cryptostegiagrandiflora*	Apocynaceae	India	Pande (2008); Farr and Rossman (2024)
*Melanommacucurbitarioideum*	*Pentaphylloidesfruticosa*	Rosaceae	China	Yuan and Barr (1994); Farr and Rossman (2024)
*Melanommadactylosporum*	Unidentified host	–	Brazil	Rehm (1901)
*Melanommadinghuense*	Unidentified host	–	China	Inderbitzin and Huang (2001)
*Melanommadistinctum*	*Pentaphylloidesfruticosa*	Rosaceae	Russia	Vasilyeva (1987)
*Melanommadrimydis*	*Drymis* sp.	Winteraceae	Brazil	Rehm (1901)
*Melanommadryinum*	*Quercus* sp.	Fagaceae	Belgium	Mouton (1900)
*Melanommadzungaricum*	*Eurotiaeversmanniana*	Amaranthaceae	Kazakhstan	Vasilyeva (1987)
*Melanommaebeni*	*Ebenusstellata*	Fabaceae	Iran	González Fragoso (1918)
*Melanommaepiphytica*	*Bambusa* sp.	Poaceae	Indonesia	Raciborski (1909); Eriksson and Yue (1998); Farr and Rossman (2024)
*Melanommagigantica*	Unidentified host	–	India	Pande (1979)
*Melanommaglumarum*	*Oryzasativa*	Poaceae	China, India, Japan and the Philippines	Watson (1971); Reinking (1919); Farr and Rossman (2024)
*Melanommagregarium*	*Populus* sp.	Salicaceae	The United States	Cash (1953); Farr and Rossman (2024)
*Melanommahalimodendri*	*Halimodendronhalodendron*	Fabaceae	Kazakhstan	Farr and Rossman (2024)
*Melanommahaloxyli*	*Haloxylonaphyllum*	Amaranthaceae	Kazakhstan	Farr and Rossman (2024)
*Melanommahelianthemi*	*Helianthemumrupifragum*	Cistaceae	Ukraine	Gucevic (1969)
*Melanommaheraclei*	*Heracleumpubescens*	Apiaceae	Ukraine	Dudka et al. (2004); Farr and Rossman (2024)
*Melanommaherpotrichum*	*Populus* sp.	Salicaceae	Luxembourg	Feltgen (1903)
*Melanommajaponicum*	*Faguscrenata*	Fagaceae	Japan	Hashimoto et al. (2017); Farr and Rossman (2024)
*Melanommajenynsii*	Unidentified host	–	The United Kingdom	Saccardo (1883)
*Melanommajuniperi*	*Juniperusvirginiana*	Cupressaceae	The United States	Fairman (1905); Cash (1953); Farr and Rossman (2024)
*Melanommalangloisii*	*Salixnigra*	Salicaceae	The United States	Farr and Rossman (2024)
*Melanommalithophilae*	*Sobolewskialithophila*	Apocynaceae	Ukraine	Dudka et al. (2004); Farr and Rossman (2024)
*Melanommalongicolle*	*Acer* sp. and *Citruslimon*	Aceraceae and Rutaceae	Italy and the United Kingdom	Saccardo (1875); Farr and Rossman (2024)
*Melanommamarathawadense*	On paper	–	India	Tilak and Kale (1970)
*Melanommamargaretae*	*Dryasoctopetala*	Rosaceae	Poland	Mulenko et al. (2008); Farr and Rossman (2024)
*Melanommamartinianum*	Unidentified host	–	New Zealand	Saccardo (1883)
*Melanommamate*	*Ilexparaguensis*	Aquifoliaceae	Argentina	Spegazzini (1908); Farr (1973); Farr and Rossman (2024)
*Melanommamedium*	*Acernegundo*, *Calligonum* sp. and *Tamarix* sp.	Sapindaceae and Tamaricaceae	Canada, Italy and the United Kingdom	Saccardo (1878); Farr (1973); Farr and Rossman (2024)
*Melanommamindorense*	*Arengamindorensis*	Arecaceae	The Philippines	Rehm (1913)
*Melanommamojunkumica*	*Haloxylonaphyllum*	Amaranthaceae	Kazakhstan	Farr and Rossman (2024)
*Melanommamoravicum*	Unidentified host	–	Slovakia	Farr and Rossman (2024)
*Melanommamutabile*	*Solanumdulcamara*	Solanaceae	Luxembourg	Feltgen (1901)
*Melanommamyricae*	*Myricagale*	Myricaceae	Sweden	Eriksson (2014); Farr and Rossman (2024)
*Melanommanigriseda*	*Fagus* sp.	Fagaceae	The United States	Fairman (1922)
*Melanommaobliterans*	Unidentified host	–	The United Kingdom	Saccardo (1883)
*Melanommaobtusissimum*	Unidentified host	–	Cuba	Farr and Rossman (2024)
*Melanommaoryzae*	*Oryzasativa*	Poaceae	Japan	Farr and Rossman (2024)
*Melanommaoxysporum*	*Quercus* sp.	Fagaceae	The United States	Hawksworth (1985)
*Melanommapanici-miliacei*	*Panicummiliaceum*	Poaceae	Siberia	Farr and Rossman (2024)
*Melanommaphilippinense*	Unidentified host	–	The Philippines	Sydow and Sydow (1914)
*Melanommapopulicola*	*Cornus* sp., *Fagussylvatica*, *Piceaabies*, *Populus* sp., *Quercus* sp. and *Sorbusaucuparia*	Cornaceae, Fagaceae, Pinaceae, Rosaceae, and Salicaceae	China, Croatia, Germany and the Netherlands	Crous et al. (2020); Farr and Rossman (2024); this study
*Melanommapraeandinum*	*Salviagilliesii*	Lamiaceae	Argentina	Spegazzini (1909); Farr (1973); Farr and Rossman (2024)
*Melanommapulveracea*	Unidentified host	–	China	Teng (1936)
*Melanommapulvis-pyrius*	*Acer* sp., *Albiziajulibrissin*, *Alhagi* sp., *Alnus* sp., *Berberis* sp., *Betula* sp., *Bupleurumfruticosum*, *Campsisradicans*, *Carpinusbetulus*, *Celtisaustralis*, *Corylus* sp., *Larixdecidua* and *Pinussylvestris*	Apiaceae, Betulaceae, Berberidaceae, Bignoniaceae, Cannabaceae, Fabaceae, Pinaceae, and Sapindaceae	Canada, Czech Republic, Japan, Poland, Russia, Scotland, Sweden and Ukraine	Mulenko et al. (2008); Hashimoto et al. (2017); Crous et al. (2020); Farr and Rossman (2024)
*Melanommapyriostictum*	Unidentified host	–	The United Kingdom	Cooke (1887)
*Melanommarhododendri*	*Rhododendron* sp.	Ericaceae	Austria, Belgium, Germany, Italy, Luxembourg, the Netherlands, Switzerland, the United Kingdom and the United States	Rehm (1881, 1906); Bommer and Rousseau (1890); Farr and Rossman (2024)
*Melanommaribis*	*Ribes* sp.	Grossulariaceae	The United States	Barr (1990)
*Melanommarubicundum*	*Lythrum* sp.	Lythraceae	Sweden	Bommer and Rousseau (1890); Farr and Rossman (2024)
*Melanommasanguinarium*	Unidentified host	–	–	Saccardo (1878)
*Melanommasaviczii*	*Thymuspseudohumillimus*	Lamiaceae	Ukraine	Dudka et al. (2004); Farr and Rossman (2024)
*Melanommascrophulariae*	*Scrophulariarupestris*	Scrophulariaceae	Ukraine	Gucevic (1967)
*Melanommasordidissimum*	*Eriobotryajaponica*	Rosaceae	Argentina	Spegazzini (1909); Farr (1973); Farr and Rossman (2024)
*Melanommasparsum*	*Abies* sp.	Pinaceae	Switzerland	Fuckel (1873)
*Melanommaspiniferum*	*Morusalba*	Moraceae	The United States	Cash (1953); Farr and Rossman (2024)
*Melanommasubandinum*	*Atriplexpamparum*	Amaranthaceae	Argentina	Cash (1953); Farr and Rossman (2024)
*Melanommasubdispersum*	*Betula* sp., and *Fagus* sp.	Betulaceae and Fagaceae	Canada, Ireland, Poland, and the United Kingdom	Conners (1967); Mulenko et al. (2008); Farr and Rossman (2024)
*Melanommasubmojunkumica*	*Haloxylonaphyllum*	Amaranthaceae	Kazakhstan	Farr and Rossman (2024)
*Melanommathespesiae*	*Thespesia* sp.	Malvaceae	India	Farr and Rossman (2024)
*Melanommatrevoae*	*Trevoatrinervia*	Rhamnaceae	Chile	Farr (1973); Farr and Rossman (2024)
*Melanommavile*	*Quercus* sp.	Fagaceae	Sweden	Fuckel (1870)
*Melanommaxylaria*e	Unidentified host	–	Brazil	Höhnel (1907)
*Monoseptellarosae*	*Rosa* sp.	Rosaceae	Uzbekistan	Wanasinghe et al. (2018)
*Muriformistrickeriarosae*	*Rosa* sp.	Rosaceae	Italy	Wanasinghe et al. (2018)
*Muriformistrickeriarubi*	*Rubus* sp.	Rosaceae	Italy	Tian et al. (2015)
*Navicellacostaricensis*	Unidentified host	–	The United States	El-Shafie et al. (2005)
*Navicelladiabola*	*Castanopsis* sp.	Fagaceae	China	Aptroot (2003)
*Navicellapallida*	*Elaeocarpus* sp.	Elaeocarpaceae	Papua New Guinea	Aptroot and Iperen (1998)
*Navicellapileata*	*Fraxinus* sp., *Salixfragilis*, *Tilia* sp. and *Quercus* sp.	Fagaceae, Malvaceae, Oleaceae, and Salicaceae	Finland, Lithuania and Norway	Fabre (1879); Holm and Holm (1988); Farr and Rossman (2024)
*Navicellaxinjiangensis*	*Lonicerahispida*, *Haloxylonammodendron*	Amaranthaceae and Caprifoliaceae	China	Yuan and Zhao (1994); Farr and Rossman (2024)
*Neobyssosphaeriaclematidis*	*Clematisvitalba*	Ranunculaceae	The United Kingdom	Phukhamsakda et al. (2020)
*Petrakiaaesculi*	*Aesculusturbinata*	Sapindaceae	Japan	Jaklitsch and Voglmayr (2017)
*Petrakiadeviata*	*Acercampestre*	Sapindaceae	Georgia and Switzerland	Watzl (1937); Gross et al. (2017)
*Petrakiaechinata*	*Acer* sp.	Sapindaceae	Italy, Slovakia, Switzerland and the United States	Sydow and Sydow (1913); Li et al. (2016a); Gross et al. (2017); Farr and Rossman (2024)
*Petrakiafagi*	*Faguscrenata*	Fagaceae	Japan	Beenken et al. (2020)
*Petrakiagreenei*	*Acersaccharinum*	Sapindaceae	The United States	Beenken et al. (2020)
*Petrakiairregularis*	*Acerpseudoplatanus*	Sapindaceae	The Netherlands and Poland	Mulenko et al. (2008); Farr and Rossman (2024)
*Petrakiajuniperi*	*Juniperus* sp.	Cupressaceae	Germany	Bedlan (2017)
*Petrakialiobae*	*Fagussylvatica*	Fagaceae	Switzerland	Beenken et al. (2020)
*Petrakiaminima*	*Fagusjaponica*	Fagaceae	Japan	Hashimoto et al. (2017); Beenken et al. (2020)
*Petrakiaparacochinensis*	*Miscanthusfloridulus*	Poaceae	China	Wong et al. (2002)
*Phragmocephalaatra*	*Rhopalostylissapida* and *Urtica* sp.	Arecaceae, Urticaceae	New Zealand and the United Kingdom	Mason and Hughes (1951); McKenzie et al. (1992)
*Phragmocephalaelegans*	*Gymnantheslucida*	Euphorbiaceae	Brazil and Cuba	Castañeda (1985)
*Phragmocephalaelliptica*	*Elaeagnus* sp., *Filipenduladenudata*, *Laurus* sp. *Quercusrobur* and *Sambucus* sp.	Adoxaceae, Elaeagnaceae, Fagaceae, Lauraceae, and Rosaceae	Canada, Russia, Ukraine and the United Kingdom	Hughes (1979); Dennis (1986); Holubtsova and Andrianova (2008); Farr and Rossman (2024)
*Phragmocephalagarethjonesii*	Unidentified host	–	China	Su et al. (2015)
*Phragmocephalaglanduliformis*	*Corticiumcoronatum*, *Piceaobovate* and *Quercus* sp.	Corticiaceae, Fagaceae, and Pinaceae	Austria	Hughes (1955); Farr and Rossman (2024)
*Phragmocephalahughesii*	Unidentified host	–	China	Wu and Zhuang (2005)
*Phragmocephalaminima*	*Abiesbalsamea* and *Fagussylvatica*	Fagaceae and Pinaceae	Canada and the United Kingdom	Mason and Hughes (1951); Conners (1967)
*Phragmocephalaprolifera*	*Populustremuloides* and *Urticadioica*	Salicaceae and Urticaceae	Belgium and Canada	Hughes (1979); Farr and Rossman (2024)
*Phragmocephalastemphylioides*	*Carya* sp., *Cistus* sp. and *Pistacialentiscus*	Anacardiaceae, Cistaceae, and Juglandaceae	Brazil, Canada, China and Italy	Hughes (1958); Zhuang (2005); Lunghini et al. (2013); Farr and Rossman (2024)
*Phragmotrichumandamanense*	*Strobilanthes* sp.	Acanthaceae	India	Bhat and Kendrick (1993)
*Phragmotrichumchailletii*	*Abies* sp. and *Picea* sp.	Pinaceae	Canada, Switzerland, Romania and the United States	Kunze and Schmidt (1823); Ginns (1986); Farr and Rossman (2024)
*Phragmotrichumkarstenii*	*Acerplatanoides*	Sapindaceae	Finland	Sutton and Pirozynski (1965)
*Phragmotrichumrivoclarinum*	*Acer* sp., *Alnus* sp. and *Salix* sp.	Betulaceae, Salicaceae, and Sapindaceae	Canada, Italy and the United Kingdom	Sutton and Pirozynski (1966); Ginns (1986); Farr and Rossman (2024)
*Phragmotrichumvassiljevae*	*Alnuskamtschatica*	Betulaceae	Russia	Melnik (1984)
*Pleotrichocladiumopacum*	Soil	–	Spain	Hernández-Restrepo et al. (2017)
*Praetumpfiaobducens*	*Fraxinusexcelsior*	Oleaceae	Austria and Sweden	Jaklitsch and Voglmayr (2017)
*Pseudobyssosphaeriabambusae*	*Bamboo* sp.	Poaceae	Thailand	Hyde et al. (2018)
*Pseudostrickeriamuriformis*	*Origanumvulgare*	Lamiaceae	Italy	Tian et al. (2015)
*Pseudostrickeriaononidis*	*Ononisspinosa*	Fabaceae	Italy	Tian et al. (2015)
*Pseudostrickeriarosae*	*Rosa* sp.	Rosaceae	Italy	Wanasinghe et al. (2018)
*Sarimanaspseudofluviatile*	Unidentified host	–	Japan	Liu et al. (2015)
*Sarimanasshirakamiense*	*Swidacontroversa*	Cornaceae	Japan	Liu et al. (2015)
*Seifertiaalpina*	*Rhododendronponticum*	Ericaceae	Austria	Beenken et al. (2020)
*Seifertiaazaleae*	*Ledumgroenlandicum*, *Leucopogoncostatus* and *Rhododendron* sp.	Ericaceae	Australia, Canada, China, Germany, Italy, Japan, the Netherlands, New Zealand, Panama, Switzerland, the United Kingdom and the United States	White and Hamilton (1935); Partridge and Morgan-Jones (2002); Farr and Rossman (2024)
*Seifertiashangrilaensis*	*Rhododendrondecorum*	Ericaceae	China	Li et al. (2016b)
*Tumulariaaquatica*	*Alnusglutinosa*, *Phragmites* sp. and *Quercus* sp.	Betulaceae, Fagaceae, and Poaceae	South Africa and the United Kingdom	Sivanesan (1984); Marvanová and Descals (1987); Farr and Rossman (2024)
*Tumulariatuberculata*	*Fagussylvatica*, *Quercus* sp.	Fagaceae	Hungary	Gönczöl (1976)
*Uzbekistanicapruni*	*Prunusarmeniaca*	Rosaceae	Russia	Hyde et al. (2020)
*Uzbekistanicarosae-hissaricae*	*Rosa* sp.	Rosaceae	Uzbekistan	Wanasinghe et al. (2018)
*Uzbekistanicavitis-viniferae*	*Vitisvinifera*	Vitaceae	Ukraine	Crous et al. (2020)
*Uzbekistanicayakutkhanika*	*Rosa* sp.	Rosaceae	Uzbekistan	Wanasinghe et al. (2018)
*Xenostigminaaceris*	*Acermacrophyllum*	Sapindaceae	The United States	Hashimoto et al. (2017)

The distribution map was prepared using MapChart programme (https://www.mapchart.net/index.html), a platform from which a personalised map of the world using different colours can be generated. The Sankey diagram was created to show the species distribution through plant host families using the free online tool SankeyMATIC by Steve Bogart (www.sankeymatic.com).

## ﻿Results

### ﻿Phylogenetic analyses

**Figure 3.**
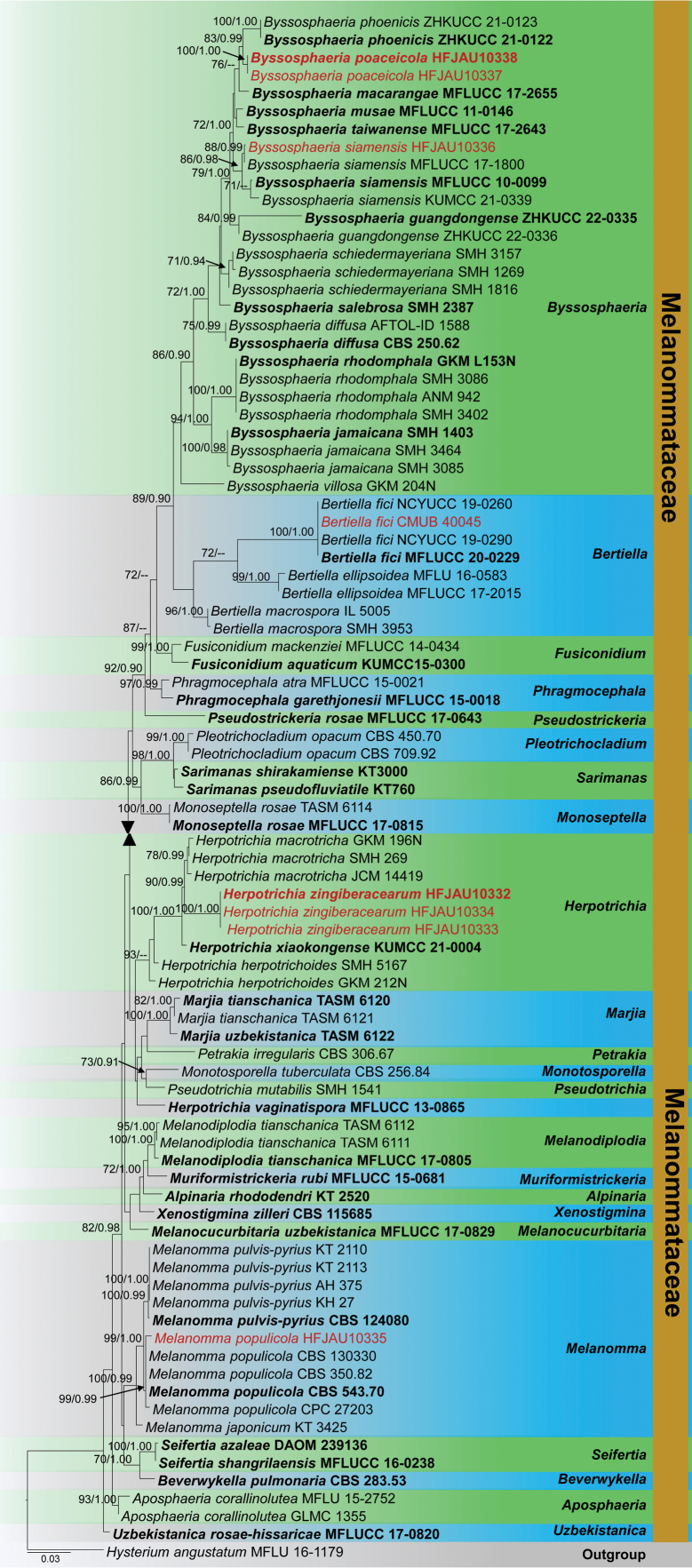
Phylogram generated from Maximum Likelihood analysis is based on combined LSU, SSU, ITS and *tef1-α* sequence data. The tree is rooted with *Hysteriumangustatum* (MFLU 16-1179). The new isolates are in red and ex-type strains are indicated in bold face. Bootstrap support values ≥ 70% from the Maximum Likelihood (ML) and Bayesian Posterior Probabilities (BYPP) values ≥ 0.90 are given above the nodes, respectively.

Phylogenetic analyses of combined LSU, SSU, ITS and *tef1-α* sequences comprised 3480 characters including gaps. *Hysteriumangustatum* (MFLU 16-1179) was used as the outgroup taxon. The RAxML analysis of the combined dataset yielded a best scoring tree (Fig. 3). The final ML optimisation likelihood value was -16620.192034. There were 35.32% undetermined characters or gaps and 1071 distinct alignment patterns. Estimated base frequencies were A = 0.247534, C = 0.235205, G = 0.272353, T = 0.244907; substitution rates AC = 1.775315, AG = 3.161447, AT = 1.766474, CG = 1.002405, CT = 11.511066, GT = 1.000; proportion of invariable sites I = 0.535605; gamma distribution shape parameter *α* = 0.513218. The Bayesian analysis has resulted in 30,000 trees after 3,000,000 generations. Bootstrap support values for ML higher than 70% and BYPP greater than 0.90 are given above each branch respectively (Fig. 3). All analyses (ML and BYPP) showed similar topologies and concurred with previous studies (Pem et al. 2019; Kularathnage et al. 2022; Gao et al. 2023).

According to the phylogeny, our collection HFJAU10336, HFJAU10337 and HFJAU10338 cluster within *Byssosphaeria* species. HFJAU10337 and HFJAU10338 cluster together and show an independent lineage, sister to *B.phoenicis* isolates (ZHKUCC 21-0122 and ZHKUCC 21-0123) with 83% ML and 0.99 BYPP statistical support. The isolate HFJAU10336 clusters with *B.siamensis* isolates (MFLUCC 10-0099, MFLUCC 17-1800 and KUMCC 21-0339) in a monophyletic clade. In addition, CBUB 40045 groups with *Bertiellafici* in a 100% ML and 1.00 BYPP supported clade. Three isolates (HFJAU10332, HFJAU10333 and HFJAU10334) cluster with *Herpotrichia* species, and show a close phylogenetic relationship with *H.melanotricha* (GKM 196N, SMH 269 and JCM 14419). As well as HFJAU10335 groups with *Melanommapopulicola* (CBS 130330, CBS 350.82, CBS 543.70 and CPC 272203) in a 99% ML and 0.99 BYPP supported clade.

### ﻿Taxonomy

#### ﻿ Bertiella

Taxon classificationFungi

(Sacc.) Sacc

98FF3456-AC6C-5527-9B97-11DD722459BF

##### Notes.

*Bertiella* was established by Saccardo and Sydow (1899) to include *B.macrospora* as the type species. The species have superficial ascomata, cylindrical-clavate asci and hyaline, 1-septate (when immature) and pale brown, 3-septate (when mature) ascospores (Tian et al. 2015; Hongsanan et al. 2020). To the present time, there are six *Bertiella* species in Species Fungorum (2024). Of them, molecular data are available only for three species. *Bertiella* species have been reported from six different plant families, Cyrillaceae, Fagaceae, Lauraceae, Moraceae, Salicaceae and Ulmaceae (Fig. 10).

#### ﻿ Bertiella fici

Taxon classificationFungi

Tennakoon, C.H. Kuo & K.D. Hyde, Fungal Diversity 108: 29 (2021)

195DBFB0-73A4-540D-93B9-4705954530BC

Index Fungorum: IF555314

Facesoffungi Number: FoF09317

Fig. 4

##### Description.

***Saprobic*** on dead leaves of *Cinnamomumverum* J. Presl (Lauraceae). **Sexual morph: *Ascomata*** 160–220 × 230–280 µm (*x̄* = 180 × 240 μm, n = 15), solitary or scattered, semi-immersed to superficial, appeared as black dots on host surface, globose to subglobose, glabrous, unilocular, ostiolate. ***Peridium*** 12–20 μm wide, thick-walled, carbonaceous, composed of several layers of brown to dark brown pseudoparenchymatous cells, cells towards the inside hyaline, arranged in a ***textura angularis***, fusing at the outside indistinguishable from the host tissues. ***Hamathecium*** comprising numerous, 1–2 µm wide, hyaline, septate, cellular pseudoparaphyses. ***Asci*** 50–60 × 7.5–8.5 μm (*x̄* = 52 × 7.8 μm, n = 20), 8-spored, bitunicate, fissitunicate, cylindrical to cylindrical-clavate, short pedicellate, apically rounded, with a distinct ocular chamber. ***Ascospores*** 14–18 × 4–5 μm (*x̄* = 15 × 4.2 μm, n = 40), overlapping, 1–2-seriate, fusiform, initially hyaline, becoming yellowish-brown at maturity, 1-septate, slightly curved, slightly constricted at the septum, guttulate, smooth-walled. **Asexual morph**: Undetermined.

**Figure 4.**
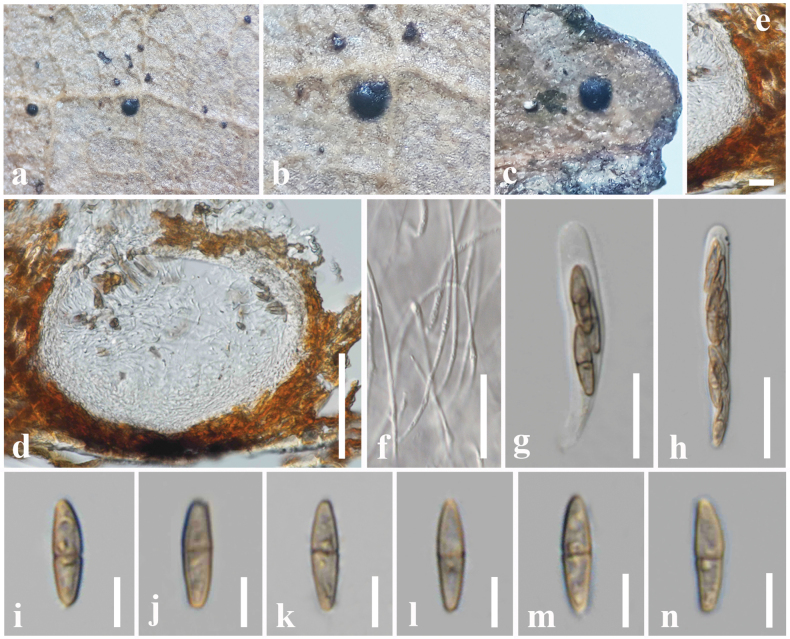
*Bertiellafici* (CMUB 40045, new host record) **a** appearance of ascomata on the host **b, c** close-up of ascomata **d** vertical section of an ascoma **e** peridium **f** pseudoparaphyses **g, h** asci **i–n** ascospores. Scale bars: 75 µm (**d**); 10 µm (**e**); 20 µm (**f–h**); 7 µm (**i–n**).

##### Material examined.

Thailand, Chiang Rai, Doi Mae Salong Mountain, on a dead leaf of *Cinnamomumverum* (Lauraceae), 15 June 2020, D. S. Tennakoon, DMS002 (CMUB 40045).

##### Known hosts.

*Cinnamomumverum* and *Ficusseptica* (Tennakoon et al. 2021; this study).

##### Known distribution.

China and Thailand (Tennakoon et al. 2021; this study)

##### Notes.

*Bertiellafici* was introduced by Tennakoon et al. (2021) from dead leaves of *Ficusseptica* in China. The morphological characteristics of our collection (CMUB 40045) tally well with the *B.fici* in having solitary or scattered, semi-immersed to superficial ascomata, cylindrical to cylindrical-clavate asci and yellowish-brown, 1-septate ascospores with overlapping size ranges (Tennakoon et al. 2021). Multi-gene phylogeny (LSU, SSU, ITS and *tef1-α*) also indicates that our collection nested with *B.fici* isolates in a 100% ML and 1.00 BYPP supported clade. This was further confirmed by having only two nucleotide differences in the LSU and SSU genes between our collection and the type of *Bertiellafici*. Thus, we conclude our collection as a new host record of *Bertiellafici* from *Cinnamomumverum*. In addition, this is the first *Bertiellafici* record from Thailand.

#### ﻿ Byssosphaeria

Taxon classificationFungi

Cooke

87E4DA73-FB1A-5EEC-9524-A5DF9E37FD7B

##### Notes.

Cooke and Plowright (1879) established *Byssosphaeria* to accommodate *B.keithii* as the type species. *Byssosphaeria* species have superficial ascomata with bright yellow, orange or red flat apices around the ostiole, with dependent hyphal appendages that merge with the subiculum below and hyaline ascospores (Tian et al. 2015; Tennakoon et al. 2018). Species have cosmopolitan distribution as saprobes in various plant substrates (e.g. dead leaves, wood). As well, *Byssosphaeria* species have been reported from 15 plant families (Fig. 10). Currently, there are 18 accepted species in Species Fungorum (2024).

#### ﻿ Byssosphaeria poaceicola

Taxon classificationFungi

Tennakoon & D.M. Hu sp. nov.

E3452DC0-B3D1-5BD7-B079-34EC81050027

Index Fungorum: IF901733

Facesoffungi Number: FoF15542

Fig. 5

##### Etymology.

Named after the host family (Poaceae) where this fungus was collected.

##### Holotype.

HFJAU10338.

##### Description.

***Saprobic*** on dead stem of *Arundopliniana* Turra (Poaceae). **Sexual morph: *Ascomata*** 550–650 × 600–800 µm (*x̄* = 610 × 715 μm, n = 10), solitary to gregarious, superficial, dark brown to black, setose, coriaceous, unilocular, globose to subglobose, non-papillate, apex rounded with an orange to yellow ostiole, ostiole central, with pore-like opening, periphysate. ***Peridium*** 30–45 μm wide, thick-walled, composed of 6–7 layers of dark brown cells, orange to yellow near ostiole, arranged in ***textura angularis***. ***Hamathecium*** 1–2.5 μm wide, comprising dense, filiform, anastomosing, septate, trabeculate pseudoparaphyses, embedded in a gelatinous matrix. ***Asci*** 165–180 × 12–15 μm (*x̄* = 171 × 13 μm, n = 20), 8-spored, bitunicate, fissitunicate, cylindrical clavate, apically rounded, long pedicellate (30–40 μm), with an indistinct ocular chamber. ***Ascospores*** 32–40 × 7–8 μm (*x̄* = 36 × 7.5 μm, n = 30), overlapping, 1–2-seriate, ellipsoid to fusiform, initially hyaline, pale brown when mature, 1-septate, constricted at the septum, slightly curved, guttulate, smooth-walled. ***A*sexual morph**: Undetermined.

**Figure 5.**
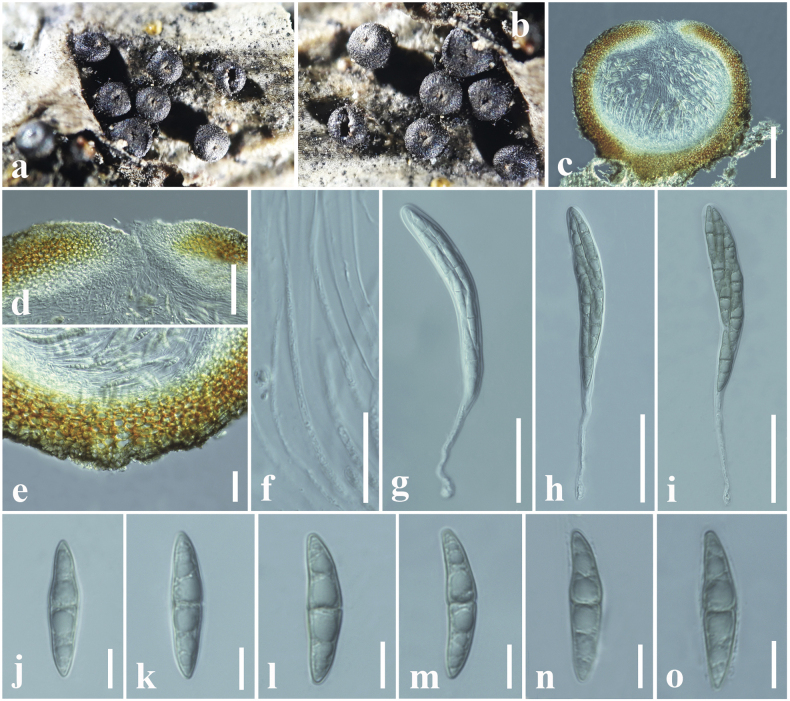
*Byssosphaeriapoaceicola* (HFJAU10338, holotype) **a, b** appearance of ascomata on the host **c** vertical section of an ascoma **d** ostiole **e** peridium **f** pseudoparaphyses **g–i** asci **j–o** ascospores. Scale bars: 200 µm (**c**); 20 µm (**d, e**); 50 µm (**f–i**); 10 µm (**j–o**).

##### Material examined.

China, Yunnan Province, Kunming, on a dead stem of *Arundopliniana* (Poaceae), 22 July 2016, D. S. Tennakoon, KDS30 (HFJAU10338, ***holotype***); *ibid*. 28 August 2016, KDS29 (HFJAU10337, ***paratype*).**

##### Notes.

In the combined LSU, SSU, ITS and *tef1-α* phylogenetic analysis, two strains of *Byssosphaeriapoaceicola* (HFJAU10337 and HFJAU10338) formed a monophyletic clade sister to *By.phoenicis* strains (ZHKUCC 21-0122 and ZHKUCC 21-012) with 83% ML and 0.99 BYPP statistical support. Morphologically, they share similarities in having superficial, dark brown to black, coriaceous, non-papillate ascomata, cylindrical clavate asci and ellipsoid to fusiform, 1-septate, pale brown ascospores (Kularathnage et al. 2022). Although *Byssosphaeriapoaceicola* can be distinguished from *By.phoenicis* in their size differences of asci (165–180 × 12–15 μm vs. 100–160 × 10–15 μm) and ascospores (32–40 × 7–8 μm vs. 25–30 × 5–7 μm) (Kularathnage et al. 2022). In addition, a comparison of the 497 nucleotides across the ITS (+5.8S) gene region of *By.poaceicola* and *By.phoenicis* shows 16 base pair differences (3.21%). It is interesting to notice that the *Byssosphaeria* species have not been collected much from Poaceae hosts, except for bamboo species (Jiang et al. 2022). Based on this finding, it appears that *Byssosphaeria* species can adapt to a variety of habitats, although there are limited studies to investigate their diversity on various hosts and regions.

#### ﻿ Byssosphaeria siamensis

Taxon classificationFungi

Boonmee, Q. Tian & K.D. Hyde, Fungal Diversity 74: 283 (2015)

7C1EBC43-69FD-5C1E-9040-10402E56F37A

Index Fungorum: IF551430

Facesoffungi Number: FoF01026

Fig. 6

##### Description.

***Saprobic*** on dead stem of *Citrustrifoliata* L. (Rutaceae). **Sexual morph: *Ascomata*** 250–400 × 300–500 µm (*x̄* = 320 × 410 μm, n = 10), solitary to gregarious, superficial, dark brown to black, setose, coriaceous, unilocular, globose to subglobose, non-papillate, apex rounded with an orange to yellow ostiole, ostiole central, with pore-like opening. ***Peridium*** 20–35 μm wide, thick-walled, composed of several layers of dark brown cells, orange to yellow near ostiole, arranged in ***textura angularis*** to ***textura prismatica***. ***Hamathecium*** 1–2.5 μm wide, comprising dense, filiform, anastomosing, septate, pseudoparaphyses, embedded in a gelatinous matrix. ***Asci*** 110–130 × 11–13 μm (*x̄* = 120 × 12 μm, n = 20), 8-spored, bitunicate, fissitunicate, cylindrical clavate, apically rounded, long pedicellate (20–35 μm), with an ocular chamber. ***Ascospores*** 30–40 × 6.5–8 μm (*x̄* = 31 × 7 μm, n = 30), overlapping, 1–2-seriate, ellipsoid to fusiform, initially hyaline, pale brown when mature, 1-septate, constricted at the septum, slightly curved, smooth-walled or verrucose. **Asexual morph**: Undetermined.

**Figure 6.**
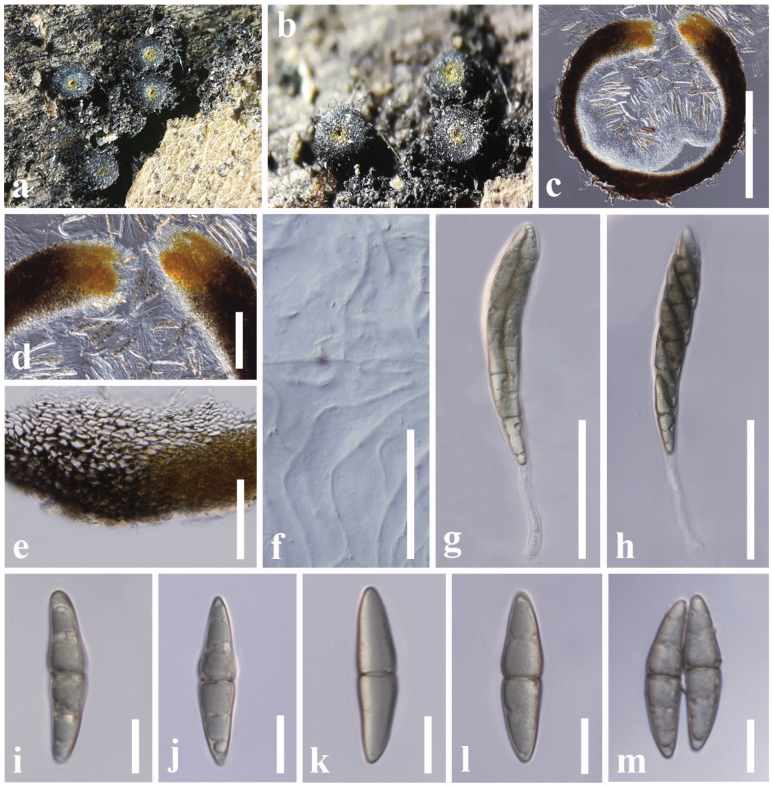
*Byssosphaeriasiamensis* (HFJAU10336, new host record) **a** appearance of ascomata on the host **b** close-up of ascomata **c** vertical section of an ascoma **d** ostiole **e** peridium **f** pseudoparaphyses **g, h** asci **i–m** ascospores. Scale bars: 200 µm (**c**); 20 µm (**d, e**); 50 µm (**f–h**); 10 µm (**i–m**).

##### Material examined.

China, Yunnan Province, Kunming, on a dead stem of *Citrustrifoliata* (Rutaceae), 27 July 2016, D. S. Tennakoon, KDS27 (HFJAU10336).

##### Known hosts.

*Citrustrifoliata* (this study).

##### Known distribution.

China, Thailand (Tian et al. 2015; Hyde et al. 2018; this study).

##### Notes.

The morphological characteristics of our collection (HFJAU10336) similar to the type of *Byssosphaeriasiamensis* in their superficial, dark brown to black, setose, non-papillate ascomata, cylindrical clavate asci and ellipsoid to fusiform, pale brown, 1-septate ascospores (Tian et al. 2015). In addition, both share overlapping size ranges of asci (110–130 × 11–13 μm vs. 112–148 × 10–16 μm) and ascospores (30–40 × 6.5–8 μm vs. 40.5–50 × 7–11 μm) (Tian et al. 2015). However, our collection is lacking a mucilaginous sheath which is present in the type species (Tian et al. 2015). According to the multi-gene phylogeny, our collection nested with *By.siamensis* isolates in 86% ML and 0.98 BYPP supported clade and close to the isolate MFLUCC 17-1800 with 88% ML, 0.99 BYPP support. Therefore, based on both morphology and phylogeny evidence, we introduce our collection as a new host record of *B.siamensis* from *Citrustrifoliata* in China.

#### ﻿ Herpotrichia

Taxon classificationFungi

Fuckel

57E2B3E7-1358-5EE8-B55F-6B26E24BFA38

##### Notes.

The diverse genus *Herpotrichia* was established by Fuckel (1868) to include two species, *H.rhenana* and *H.rubi*, but without designating a type species. Thus, Bose (1961) assigned *H.rhenana* as the lectotype. Subsequently, Holm (1979) assigned *H.herpotrichoides* (synonymous with *H.rubi*) as the generic type (Cannon 1982). The species have erumpent to superficial ascomata, clavate to cylindrical asci with hyaline to pale brown, 1-septate ascospores (Sivanesan 1984; Tian et al. 2015). The asexual morph is pyrenochaeta-like with or without setae on the surface of the pycnidia (Sivanesan 1984; Hongsanan et al. 2020). *Herpotrichia* species have been reported as saprobes, mostly in dead wood substrates in 34 plant families (Fig. 10).

#### ﻿ Herpotrichia zingiberacearum

Taxon classificationFungi

Tennakoon & D.M. Hu sp. nov.

182625B3-2213-54CD-BA9D-1F3D47F5D535

Index Fungorum: IF901734

Facesoffungi Number: FoF15543

Fig. 7

##### Etymology.

Named after the host family (Zingiberaceae) where this fungus was collected.

##### Holotype.

HFJAU10332.

##### Description.

***Saprobic*** on dead stem of *Hedychiumcoronarium* J. Koenig (Zingiberaceae). **Sexual morph: *Ascomata*** 250–350 × 240–320 µm (*x̄* = 298 × 262 μm, n = 10), solitary to clustered, superficial, dark brown to black, setose, coriaceous, unilocular, globose to subglobose, rounded apex broadly cap-like, ostiolate. ***Peridium*** 15–25 μm wide, thick-walled, composed of 4–5 layers of dark brown to black cells, arranged in ***textura angularis***. ***Hamathecium*** 1–3 μm wide, comprising dense, filiform, anastomosing, septate, branched pseudoparaphyses, embedded in a gelatinous matrix. ***Asci*** 85–98 × 10–14 μm (*x̄* = 94 × 12 μm, n = 20), 8-spored, bitunicate, fissitunicate, cylindrical clavate, apically rounded, short pedicellate, with an ocular chamber. ***Ascospores*** 25–30 × 5–6 μm (*x̄* = 28 × 5.2 μm, n = 30), overlapping, 1–2-seriate, fusoid with narrowly rounded ends, hyaline, 1-septate, straight to slightly curved, constricted at the septum, with large guttules, surrounded by an expanded gelatinous sheath pointed at both ends, 2.5–4 μm wide, smooth-walled. **Asexual morph**: Undetermined.

**Figure 7.**
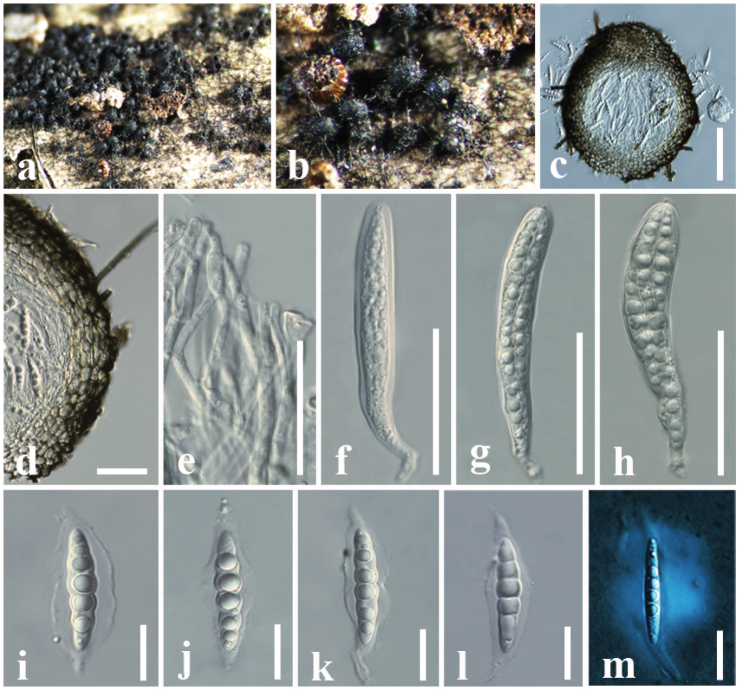
*Herpotrichiazingiberacearum* (HFJAU10332, holotype) **a, b** appearance of ascomata on the host **c** vertical section of an ascoma **d** peridium **e** pseudoparaphyses **f–h** asci **i–l** ascospores **m** ascospore stained in Indian ink showing a mucilaginous sheath. Scale bars: 100 µm (**c**); 20 µm (**d**); 50 µm (**e–h**); 15 µm (**i–m**).

##### Material examined.

China, Yunnan Province, Kunming, on a dead stem of *Hedychiumcoronarium* (Zingiberaceae), 20 July 2016, D. S. Tennakoon, KDS12 (HFJAU10332, ***holotype***); *ibid*. 21 August 2016, KDS13 (HFJAU10333, ***paratype***); *ibid*. 25 August 2016, KDS18 (HFJAU10334, ***paratype***).

##### Notes.

*Herpotrichiazingiberacearum* is isolated from the dead stem of *Hedychiumcoronarium* (Zingiberaceae). The newly-generated sequences *H.zingiberacearum* (LSU, SSU, ITS and *tef1-α*) formed a monophyletic clade closely related to *H.macrotricha* with 90% ML and 0.99 BYPP statistical support. Morphologically, they share similarities in having dark brown to black, setose, coriaceous ascomata, cylindrical clavate asci and hyaline, 1-septate ascospores (Mugambi and Huhndorf 2009). However, *H.zingiberacearum* can be distinguished from *H.macrotricha* in their smaller asci (85–98 × 10–14 μm vs. 115–145 × 11–13 μm) and ascospores (25–30 × 5–6 μm vs. 30–35 × 4–6 μm) (Tanaka and Hosoya 2006). On the other hand, *H.zingiberacearum* (HFJAU10332) differs from *H.macrotricha* (GKM 196N) by a comparison of the 694 nucleotides across the *tef1-α* gene region which shows 20 base pair differences (3.02%). This finding addresses future studies to explore the diversity of *Herpotrichia* species in different geographic regions and host plants, as these species have not previously been described from Zingiberaceae hosts (Table 2).

#### ﻿ Melanomma

Taxon classificationFungi

Nitschke ex Fuckel

F5BB76BA-9FB8-5A28-B3BB-AE26B5A69390

##### Notes.

*Melanomma* was validly established by Fuckel (1870) with *M.pulvis-pyrius* as the type species. These species have cosmopolitan distribution worldwide and characterised in having carbonaceous ascomata and hyaline or brown, 2–3-septate ascospores (Tian et al. 2015; Crous et al. 2020). The asexual morph can be either coelomycetes or hyphomycetes (Hyde et al. 2011). Currently, 94 species are accepted in this genus (Species Fungorum 2024) and have been reported from 39 plant families (Fig. 10).

#### ﻿ Melanomma populicola

Taxon classificationFungi

Crous & R.K. Schumach, Fungal Systematics and Evolution 6: 201 (2020)

61DD9169-8B6F-5F38-893C-C3049222A7AA

Index Fungorum: IF552757

Facesoffungi Number: FoF2887

Fig. 8

##### Basionym.

*Aposphaeriapopulina* Died., Krypt.-Fl. Brandenburg (Leipzig) 9: 206 (1912).

##### Synonym.

*Melanommapopulinum* (Died.) Phukhams. & K.D. Hyde [as ‘*populina*’], Fungal Diversity 83: 49. 2017.

##### Description.

***Saprobic*** on dead stem of *Fagussylvatica* L. (Fagaceae). **Sexual morph: *Ascomata*** 130–200 × 200–300 µm (*x̄* = 150 × 255 μm, n = 15), solitary or scattered, immersed, erumpent through host surface, black, multi-loculate, globose to subglobose, ostiolate. ***Peridium*** 10–15 μm wide, thick-walled, carbonaceous, composed of several layers of light brown to dark brown pseudoparenchymatous cells, cells towards the inside hyaline, arranged in a ***textura angularis***, fusing at the outside indistinguishable from the host tissues. ***Hamathecium*** comprising numerous, 1–2 µm wide, hyaline, septate, filiform pseudoparaphyses. ***Asci*** 80–110 × 6–8 μm (*x̄* = 96 × 7 μm, n = 20), 8-spored, bitunicate, fissitunicate, cylindrical, apically rounded, short pedicellate with furcate end, with an indistinct ocular chamber. ***Ascospores*** 13–17 × 4–5.2 μm (*x̄* = 15 × 4.8 μm, n = 35), overlapping, 1–2-seriate, ellipsoid, initially hyaline, becoming light brown at maturity, 3-septate, straight to slightly curved, slightly constricted at the septa, guttulate, smooth-walled. **Asexual morph**: See Crous et al. (2020).

**Figure 8.**
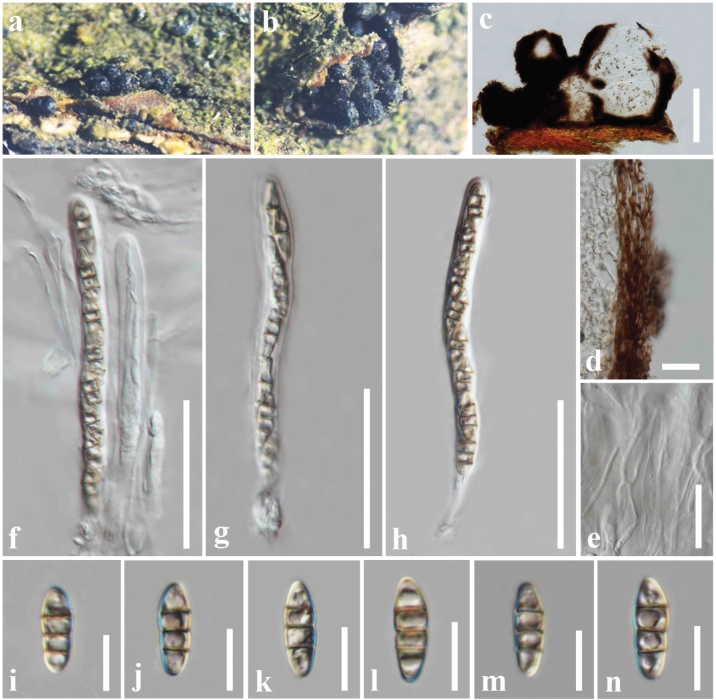
*Melanommapopulicola* (HFJAU10335, new host record) **a** appearance of ascomata on the host **b** close-up of ascoma **c** vertical section of an ascoma **d** peridium **e** pseudoparaphyses **f–h** asci **i–n** ascospores. Scale bars: 75 µm (**c**); 10 µm (**d**); 40 µm (**e–h**); 10 µm (**i–n**).

##### Material examined.

China, Kunming, on a dead stem of *Fagussylvatica* (Fagaceae), 25 July 2016, D. S. Tennakoon, KDS25 (HFJAU10335).

##### Known hosts.

*Populuscanadensis*, *Piceaabies*, *Quercus* sp., *Sorbusaucuparia* (Crous et al. 2020; this study).

##### Known distribution.

China, Germany, The Netherlands (Crous et al. 2020; this study).

##### Notes.

*Melanommapopulicola* was introduced by Diedicke (1912) as *Aposphaeriapopulina*, which was a phoma-like species collected from *Populuscanadensis*. An epitype for *A.populina* was established by De Gruyter et al. (2013). Tibpromma et al. (2017) synonymised *A.populina* under *Melanomma* and erected as *M.populina*. Subsequently, this was validated by Crous et al. (2020) and erected as *M.populicola*. Morphological characteristics of our collection (HFJAU10335) fit well with the *M.populicola* in having black, globose to subglobose ascomata, cylindrical, apically rounded asci and ellipsoid, 3-septate, light brown ascospores. In addition, there are overlapping size ranges of asci (80–110 × 6–8 μm vs. 91–106 × 6.5–7.5 μm) and ascospores (13–17 × 4–5.2 μm vs. 15.1 × 5 μm) (Crous et al. 2020). Multi-gene phylogeny also shows that our collection groups with *M.populicola* isolates in a 99% ML and 0.99 BYPP supported clade. Therefore, we introduce our collections as a new host record of *M.populicola* from *Fagussylvatica*.

### ﻿Geographical distribution and host associations of melanommataceous species

**Figure 9.**
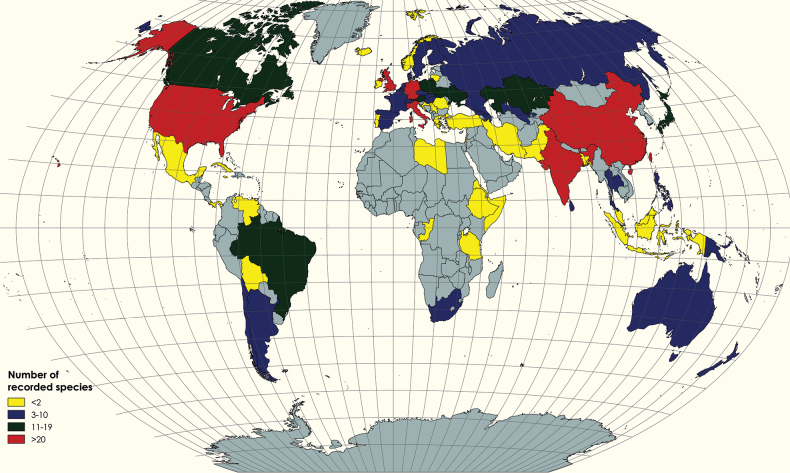
Distribution of so far reported species in Melanommataceae worldwide. Colour gradient shows the number of recorded species from lowest (yellow) to highest (red) and no records (grey).

Based on the data collected, it appears that the members of Melanommataceae are widely distributed around the world, comprising subtropical, tropical and temperate regions, such as Austria, Australia, Brazil, Canada, Finland, Germany, Japan, India, Thailand, Papua New Guinea, South Africa, Ukraine and the United States). The highest number of species have been reported from the United States (48 species). This is followed by China (35 species), Italy (30 species), India (25 species), Germany (23 species) and the United Kingdom (22 species) (indicated by red areas, Fig. 9). Moreover, Japan (17 species), Brazil (16 species), Ukraine (16 species), Canada (15 species), Kazakhstan (13 species), Poland (13 species) and Austria (11 species) are indicated by green areas (Fig. 9). Twenty-four countries have the species number range between 3 and 10, for instance Argentina, Russia and Sweden (10 species), Uzbekistan (9 species), Australia, France and Switzerland (8 species), Chile (7 species), Luxembourg, New Zealand, the Philippines and Thailand (6 species), Finland (5 species), Belgium and South Africa (4 species) and three species in nine countries (Azerbaijan, Czechia, Denmark, Georgia, Hungary, Papua New Guinea, Slovakia, Spain and Sri Lanka) (indicated by blue areas, Fig. 9). In addition, 36 countries reported two or a single species (indicated by yellow areas, Fig. 9). This may be because those countries have very limited taxonomic investigations on melanommataceous species. Consequently, it would be important to conduct more comprehensive collections, carry out more taxonomic studies and identify the species in those poorly-explored countries (e.g. African continent). In Asia, the highest number of melanommataceous species have reported from China, India and Thailand (Fig. 9), while Austria, Germany, Italy, Poland and the United Kingdom in Europe also have high numbers.

Some species exist over multiple continents, while others appear to have limited distribution and are currently reported from one or a few countries (Fig. 9). Nine species of Melanommataceae are intercontinental and occur on more than five countries. Some highly diverse species include *Aposphaeriapulviscula*, *Camposporiumcambrense*, *C.pellucidum*, *Herpotrichiaherpotrichoides*, *H.nigra*, *H.quinqueseptata*, *Melanommapulvis-pyrius*, *M.rhododendri* and *Seifertiaazaleae* (Table 2). As well, nine species have been reported from four countries (*Byssosphaeriajamaicana*, *By.rhodomphala*, *Camposporiumantennatum*, *Herpotrichiamacrotricha*, *Melanommaglumarum*, *M.subdispersum*, *Petrakiaechinata*, *Phragmocephalaelliptica* and *Phragmotrichumchailletii*) and nine species from three countries (*Aposphaeriaeragrostidis*, *A.freticola*, *A.mediella*, *Camposporiumhyderabadense*, *Melanommamedium*, *M.populicola*, *Navicellapileata*, *Phragmocephalastemphylioides* and *Phragmotrichumrivoclarinum*). In contrast, the distribution of 313 species has been limited to two or to one country, based on the current information. We believe that the distribution of those species may be much higher with future collections and taxonomical investigations.

When focusing on the host association of melanommataceous species, most of have been discovered in decaying wood or submerged woody substrates (e.g. *Aposphaeriacorallinolutea*, *A.rudis*, *Asymmetricosporacalamicola*, *Bertiellaellipsoidea*, *Byssosphaeriajuniperi*, *Calyptronectriaargentinensis*, *Camposporiumchinense*, *C.marylandicum*, *Herpotrichiaalpincola*, *Marjiauzbekistanica*, *Melanommadinghuense*, *Phragmocephalagarethjonesii* and *Sarimanaspseudofluviatile*) (Table 2). Some species have been recorded from decaying leaves (e.g. *Bertiellafici*, *Byssosphaeriamusae*, *Phragmocephalaelegans*). As well, it is noteworthy to mention that some species have been recorded from soil (e.g. *Herpotrichiagelasinosporoides*, *H.striatispora* and *Pleotrichocladiumopacum*). In addition, *Aposphaeriaramalinae* has been collected from a lichen species (*Ramalinaimplectens*) in France (Pitard and Harmand 1911) and *Exosporiellafungorum* from a mushroom species (*Thelephora* sp. fibre vase or earth fan mushroom) in Sweden (Karsten 1892).

**Figure 10.**
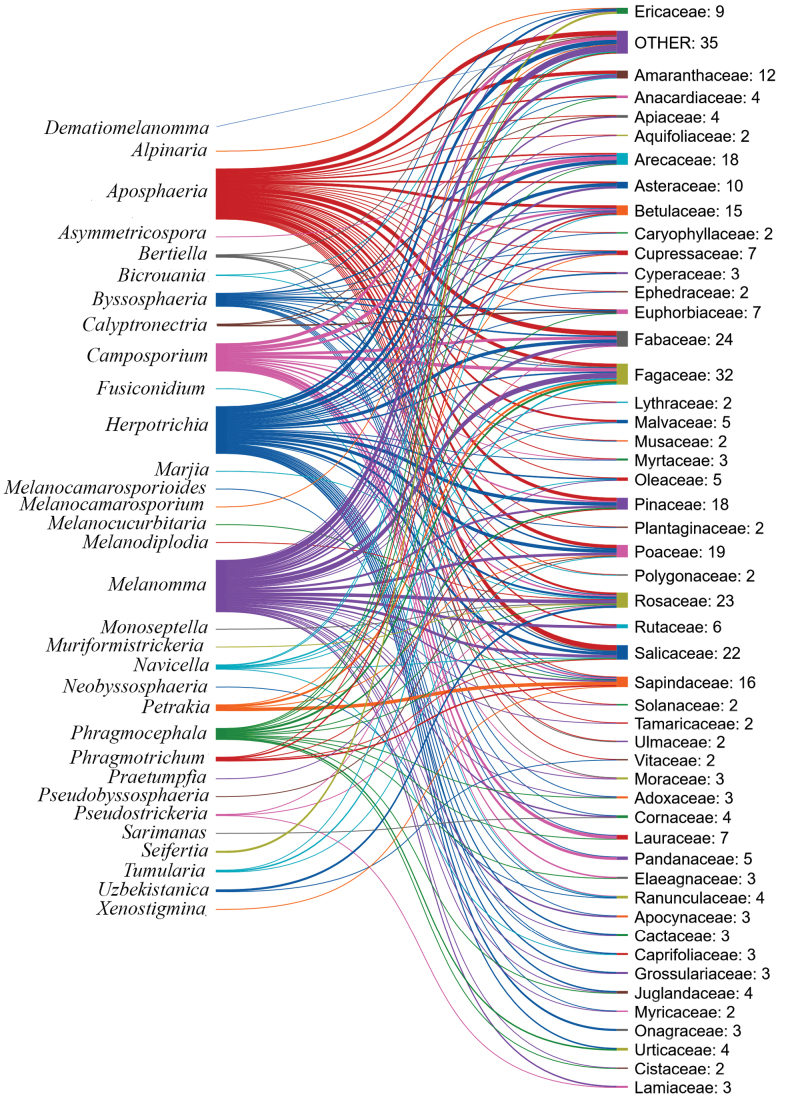
The melanommataceous species distribution through plant host families.

According to the host associations, some species of Melanommataceae are highly diverse and have been collected from more than 10 host species (e.g. *Camposporiumantennatum*, *C.japonicum*, *C.pellucidum*, *Herpotrichiamacrotricha*, *H.nigra* and *Melanommapulvis-pyrius*), while some are from more than five host species (e.g. *Camposporiumcambrense*, *Herpotrichiaherpotrichoides*, *Melanommapopulicola* and *Phragmocephalaelliptica*). The highest number of melanommataceous species have reported from the plant family Fagaceae (32 species). The most common Fagaceae hosts are *Fagus* spp. (e.g. *F.crenata*, *F.sylvatica*) and *Quercus* species (e.g. *Q.germana*). This is followed by Fabaceae (24 species), Rosaceae (23 species), Salicaceae (22 species), Poaceae (19 species), Pinaceae and Arecaceae (18 species), Sapindaceae (16 species), Betulaceae (15 species), Amaranthaceae (12 species), Asteraceae (10 species), Ericaceae (9 species), Cupressaceae, Euphorbiaceae and Lauraceae (7 species), Rutaceae (6 species) and Malvaceae, Oleaceae and Pandanaceae (5 species) (Fig. 10). In addition, a range of 2–5 species have recorded from 30 plant families and single melanommataceous species have reported from 35 plant families. Thus, up to date, the species of the family have been reported from 82 plant families. However, some species have been collected from unidentified hosts and, thus, their host association remained unresolved (e.g. *Aposphaeriaanomala*, *A.pakistanica*, *Bertiellaellipsoidea*, *B.gelatinosa*, *Camposporiumappendiculatum* and *C.chinense*). The host specificity for most of the species has not yet been clarified, as they have been recorded from various plant families. Though, it was noted by Jaklitsch and Voglmayr (2017) that *Praetumpfiaobducens* may be host specific to *Fraxinus* species.

## ﻿Discussion

The members of Melanommataceae have been well-studied in last two decades, leading to many exciting discoveries (17 new genera and 76 new species), which may be mostly linked to the progress made in the DNA sequence data. The initial classification of these species was primarily based on morphological characteristics (e.g. globose or depressed ascomata, trabeculate pseudoparaphyses, fissitunicate asci, pigmented ascospores) associated with hand drawings (Saccardo 1883; Winter 1885; Morgan 1904; Spegazzini 1909; Shear 1941). However, morphology-based identification is challenging since most species can share similar characteristics, which may lead to misinterpretations. Thus, a combination of DNA-based molecular phylogeny and morphological traits has become a widely accepted tool for current fungal classifications (Tian et al. 2015; Hashimoto et al. 2017; Jaklitsch and Voglmayr 2017; Purahong et al. 2017; Kularathnage et al. 2022). Mostly, the large subunit (28S, LSU), small subunit (18S, SSU), internal transcribed spacers (ITS1–5.8S–ITS2), translation elongation factor 1 gene (*tef1-α*) and RNA polymerase second largest subunit (*rpb2*) molecular markers have been used in current phylogenetic analyses of Melanommataceae (Tian et al. 2015; Tennakoon et al. 2018; Pem et al. 2019; Kularathnage et al. 2022).

Melanommataceae genera are highly varied, both morphologically and phylogenetically. Of them, some are highly diverse with numerous species (e.g. *Aposphaeria*: 83 species, *Melanomma*: 81 species, *Herpotrichia*: 61 species, *Camposporium*: 25 species, *Byssosphaeria*: 18 species and *Petrakia*: 10 species), while some have fewer species (*Phragmocephala*: 9 species, *Bertiella*: 6 species, *Navicella*: 5 species, *Phragmotrichum*: 5 species, *Uzbekistanica*: 4 species, *Calyptronectria*: 3 species, *Pseudostrickeria*: 3 species, *Seifertia*: 3 species, *Marjia*: 2 species, *Muriformistrickeria*: 2 species, *Sarimanas*: 2 species, and *Tumularia*: 2 species). In addition, some genera are monotypic and need more collections for their expansion (e.g. *Alpinaria*, *Asymmetricospora*, *Bicrouania*, *Dematiomelanomma*, *Exosporiella*, *Fusiconidium*, *Mamillisphaeria*, *Melanocamarosporioides*, *Melanocamarosporium*, *Melanocucurbitaria*, *Melanodiplodia*, *Monoseptella*, *Neobyssosphaeria*, *Pleotrichocladium*, *Praetumpfia*, *Pseudobyssosphaeria* and *Xenostigmina*). However, it is noteworthy to mention that some genera currently lack molecular data and, thus, their phylogenetic position is uncertain (e.g. *Asymmetricospora*, *Bicrouania*, *Calyptronectria*, *Exosporiella*, *Mamillisphaeria* and *Navicella*) (Hongsanan et al. 2020). As well, only two species of *Aposphaeria* have molecular data out of the 84 species listed in the Species Fungorum (2024). Consequently, to resolve their phylogenetic placements, further collections and investigations are essentially required.

In this study, we introduced two new species and three new host records collected from China and Thailand. The new species, *Byssosphaeriapoaceicola* and *Herpotrichiazingiberacearum* can be distinguished from related species in their morphology and DNA molecular data. The morphological characteristics of the new host records strongly tally with their type species and phylogeny analyses also provide evidence for their placements. These new host records also demonstrate their adaptability to a broad range of habitats and there could be many more. Thus, many fungal species and host associations are waiting for us and we should undertake further explorations.

## Supplementary Material

XML Treatment for Bertiella

XML Treatment for Bertiella fici

XML Treatment for Byssosphaeria

XML Treatment for Byssosphaeria poaceicola

XML Treatment for Byssosphaeria siamensis

XML Treatment for Herpotrichia

XML Treatment for Herpotrichia zingiberacearum

XML Treatment for Melanomma

XML Treatment for Melanomma populicola

## ﻿Additional information

### Author ORCIDs

Danushka S. Tennakoon https://orcid.org/0000-0003-2306-1255

Kasun M. Thambugala https://orcid.org/0000-0002-6210-0504

Nimali I. de Silva https://orcid.org/0000-0002-1577-280X

Nakarin Suwannarach https://orcid.org/0000-0002-2653-1913

Dian-Ming Hu https://orcid.org/0000-0002-4750-2871

### Data availability

All of the data that support the findings of this study are available in the main text.
